# The gap between guidelines and clinical practice in cancer-related fatigue management: a mixed-methods systematic review

**DOI:** 10.1007/s11764-025-01866-6

**Published:** 2025-07-18

**Authors:** Marijn Witziers, Anne Looijmans, Ning Jia, Mariët Hagedoorn

**Affiliations:** Department of Health Psychology, University Medical Center Groningen, University of Groningen, Groningen, the Netherlands

**Keywords:** Cancer-related fatigue, Guideline adherence, Symptom Management, Cancer care

## Abstract

**Purpose:**

Cancer-related fatigue (CRF) is the most prevalent and disruptive side effect of cancer and its treatment, significantly impairing patients’ quality of life. While guidelines mandate periodic screening, in-depth assessments, and non-pharmacological treatments for fatigue, the adherence of healthcare professionals (HCPs) to these guidelines is unknown. Therefore, this review aims to synthesize existing knowledge on HCPs’ adherence to CRF guidelines.

**Methods:**

A systematic review was conducted, encompassing searches across six databases and Google Scholar. Studies reporting on CRF management, barriers to CRF care experienced by HCPs, or interventions aimed at improving CRF management were included. A narrative synthesis approach was employed to analyze findings.

**Results:**

Of the 7039 identified publications, seventy-seven studies met the inclusion criteria. Findings reveal notable discrepancies between guideline recommendations and clinical practice. Specifically, fatigue screening was inconsistently performed across healthcare settings, comprehensive fatigue assessments were scarce, and pharmacological interventions were commonly used to alleviate fatigue. Barriers such as time constraints and insufficient knowledge hindered optimal CRF management.

**Conclusion:**

Findings of this review suggest that HCPs do generally not adhere to clinical guidelines for fatigue management.

**Implications for Cancer Survivors:**

There is ample room to improve CRF management, with systematic changes essential to addressing barriers to effective fatigue management. As part of these changes, enhancing HCP education can help to frame CRF as a manageable, multifaceted symptom. These improvements will contribute to better fatigue communication, recognition, timely support, and a more coordinated approach to care for patients affected by this debilitating condition.

**Supplementary Information:**

The online version contains supplementary material available at https://doi.org/10.1007/s11764-025-01866-6.

## Introduction

Cancer-related fatigue (CRF) is a widespread and debilitating symptom that substantially reduces the quality of life (QoL) of cancer patients and survivors [1–4]. Recognizing its extensive impact, numerous national and international organizations have established clinical guidelines to aid healthcare professionals (HCPs) in the management of CRF [5–8]. Since the initial publication of these guidelines in the year 2000, their core structure has remained largely unchanged, emphasizing three fundamental stages of CRF management: screening, assessment, and treatment. Periodic screening for the presence of fatigue is recommended, preferably using a numeric rating scale. Following a positive screening result, a thorough assessment of fatigue ought to be conducted to ascertain its severity and to identify potential physical or psychosocial causes. In the absence of apparent underlying causes other than cancer and its treatment, patients should receive an appropriate course of treatment for their CRF, which may encompass energy management, psychoeducation, psychosocial counselling, physical activity, cognitive behavioral therapy, or a combination of these approaches.

Studies published prior to or shortly following the introduction of the CRF guidelines accentuate the lack of uniformity in CRF care, thereby underscoring the necessity for CRF guidelines. A review from 2004 indicates that many patients experiencing fatigue had never discussed it with an HCP, only a minority had received advice regarding their fatigue, and both patients and HCPs agreed that resting was the most recommended strategy for alleviating fatigue [9]. Given these previous shortcomings in CRF management, it is imperative to evaluate whether HCPs have been adhering to the CRF guidelines since their publication.

An exploratory examination of the literature published since the dissemination of the guidelines reveals considerable variability in guideline adherence across clinical settings. While certain studies reveal that fatigue is commonly discussed among cancer patients and HCPs and that patients are regularly screened for the presence of fatigue, other studies imply that such discussions and screening practices are scarce [10–15]. Furthermore, some studies suggest that a majority of patients get treated for CRF, whereas others highlight a discrepancy between the widespread availability of CRF interventions and the limited number of patients who actually receive them [16–19]. It should be noted that these preliminary findings are not comprehensive, and no study to date has systematically assessed and summarized HCPs’ adherence to CRF guidelines following their publication. Therefore, the current systematic review synthesizes existing knowledge on HCPs’ adherence to CRF guidelines. Additionally, this review examines CRF management from the differing perspectives of HCPs and patients, identifies barriers and facilitators to CRF management and guideline adherence encountered by HCPs, and explores interventions aimed at improving compliance with the CRF guidelines.

## Methods

The present systematic review is reported in accordance with the 2020 Preferred Reporting Items for Systematic Review and Meta-Analyses (PRISMA) guidelines [20]. The protocol of this review was registered in advance within the International Prospective Register of Systematic Reviews (PROSPERO) database (ID: CRD42023412279).

### Study eligibility and search strategy

The current review sought to identify studies: (1) reporting on outcomes related to HCPs’ adherence to CRF guidelines, comprising guideline usage and the guideline’s key stages of CRF management—screening, assessment, and treatment—as well as barriers and facilitators to CRF management faced by HCPs. Additional outcomes of interest for CRF management were identified in the literature and subsequently incorporated into this review, including fatigue information, advice, communication, and documentation. (2) Focusing on CRF management provided by doctors, nurses, and other medical specialists primarily responsible for patients’ cancer care. Additionally, general practitioners (GPs) were included, recognizing that in some healthcare systems GPs may hold primary responsibility for patients in palliative care and those in aftercare. Furthermore, GPs can play a critical role in symptom management throughout a patient’s entire treatment trajectory. All aforementioned HCPs are collectively referred to as clinical care providers within this review. (3) Conducted from the year 2000 onwards, coinciding with the first publication of the CRF guidelines. When the study year was unspecified, authors were contacted for clarification. The full inclusion and exclusion criteria and the definitions of all considered outcomes are described in Table 1.

**Table 1:** The inclusion and exclusion criteria for the present review, structured according to the PICOS format

Inclusion criteria	Exclusion criteria
*Population*	
Healthcare professionals • HCPs primarily responsible for cancer care, such as: ○ Oncologists, nurses, physician assistants, and GPs. Patients • Adult (aged ≥ 18 years) cancer patients and survivors. Studies in which the target population as described above constituted a subset of the total study population were eligible if: 1) The data pertinent to the target population could be extracted, or 2) The target population accounted for ≥ 80% of the total study sample.	Healthcare professionals • HCPs primarily involved in supportive care for cancer patients, such as: ○ Physical therapists, psychologists, and social workers. Patients • Patients who never had cancer. • Underaged patients (< 18 years).
*Intervention*	
Solely applicable for experimental studies: • Studies that provided baseline and intervention data for one or more of the outcomes of interest. • Interventions that were conducted in settings where they directly impacted the target population of interest. Studies in which the intervention did not meet the inclusion criteria were still eligible when they reported baseline data on one or more outcomes of interest.	Solely applicable for experimental studies: • Studies that reported on outcomes solely after the intervention had been applied, without baseline data for comparison. • Interventions that were administered in settings where they did not directly impact the target population.
*Comparator*	
Solely applicable for experimental studies: • Comparators, including active interventions or care-as-usual, present in settings where they directly impacted the population of interest. Studies in which the comparator did not meet the inclusion criteria were still eligible when they reported baseline data on one or more outcomes of interest.	Solely applicable for experimental studies: • Comparators provided in settings that did not have a direct impact on the target population.
*Outcome*	
• All reported quantitative and qualitative findings on outcomes related to guideline usage or the key stages of the CRF management guidelines. ○ Guideline usage: Application of the CRF guidelines in practice; measures include the prevalence and frequency of guideline adherence or usage, as well as descriptions of internal implementation. ○ Screening: Determining the potential presence of fatigue; measures include screening prevalence and frequency and details of the screening instruments employed. ○ Assessment: In-depth evaluation of fatigue and the identification and treatment of underlying causes; measures include the prevalence of assessment and descriptions of the methods used. ○ Treatment: Provision of interventions aimed at alleviating CRF; measures include the prevalence of treatment discussions, provided treatment types, and treatment availability. • All reported quantitative and qualitative outcomes related to HCPs’ experienced barriers or facilitators to CRF management or CRF guideline usage. ○ Barriers: Factors experienced by HCPs that hinder effective CRF management or CRF guideline adherence or implementation; including systemic, organizational, personal, and patient-related factors. ○ Facilitators: Factors experienced by HCPs that support or enhance effective CRF management or CRF guideline adherence or implementation; including systemic, organizational, personal, and patient-related factors. • All reported quantitative and qualitative findings on the additionally identified outcomes of interest for CRF management: information, communication, advice, and documentation. ○ Information: Provision of patient information or education regarding CRF; measures include the prevalence of information provision, as well as the scope and quality of the information provided. ○ Advice: Provision of recommendations aimed at alleviating fatigue without involving specific interventions or treatments; measures include the prevalence and type of advice offered. ○ Communication: Any discussion or exchange of CRF information not categorizable under other outcomes; measures include the prevalence and frequency of communication, along with the conversation’s initiator and topics. ○ Documentation: Recording of all information related to fatigue management in patients’ clinical records; measures include documentation of fatigue presence, severity, and associated care.	• Outcomes related to fatigue management outside the context of cancer care. • Barriers and facilitators to CRF management encountered by patients.
*Study design*	
• Empirical studies using: ○ Quantitative designs, including cohort studies, cross-sectional studies, and interventional studies. ○ Qualitative designs, including interview studies, focus group studies, and ethnographic approaches. ○ Mixed-methods studies combining quantitative and qualitative approaches.	• Case-control studies, case-series, and case reports. • Systematic reviews and meta-analyses.
*Publication date and language*	
• Studies that were conducted from the year 2000 onwards. ○ Studies without a reported study year were eligible if published from the year 2002 onwards. • All languages were considered.	• Studies that were conducted prior to the year 2000.

*CRF*, cancer-related fatigue; *GP*, general practitioner; *HCP*, healthcare professional

A systematic search was performed in MEDLINE, Embase, Web of Science, Scopus, CINAHL, and CENTRAL databases, covering publications up to April 5, 2023. The search strategy, developed in collaboration with a medical librarian specialized in systematic reviews, was originally developed for the MEDLINE database and later adapted for each database’s specific requirements, as detailed in Supplementary Materials File 1. Search alerts were enabled across all databases to identify newly published studies meeting the search strategy. Relevant publications retrieved through these alerts were included up to October 25, 2023, prior to the start of the data analysis. Additionally, Google Scholar was used to complement the database searches, as it allows searches within full-text articles [21]. All publications from the first twenty pages of search results were considered, while those of pages twenty-one to thirty were screened for relevance based on title and abstract. Finally, backward reference screening was conducted on relevant systematic reviews identified through the search strategy.

### Screening and data extraction

Identified publications were deduplicated in EndNote according to Bramer’s method [22]. The screening process, facilitated by Rayyan software, consisted of two screening phases, each phase independently carried out by two reviewers (MW & NJ) [23]. In the first stage, studies were screened based on their title and abstract, followed by a full-text screening in the second phase. When a full-text could not be retrieved, the corresponding authors were contacted to request a copy. Disagreements between both reviewers were resolved by finding consensus. If consensus could not be reached in the first stage, the study was moved to the succeeding phase. In the second stage, any remaining disagreements were addressed through discussions with the third and fourth reviewers (AL & MH). Prior to each screening phase, two calibration sessions were conducted to ensure clarity of the inclusion and exclusion criteria and to standardize reviewers’ judgements.

The first reviewer (MW) extracted data for all included studies using pre-developed data extraction tables in Microsoft Excel, with the second reviewer (NJ) verifying correct extraction of the data for 25% of the studies published in English. Data were extracted exactly as reported in the original studies, without any alterations, and missing data were left as empty fields with no assumptions or imputations made. Any discrepancies in the extracted data between both reviewers were resolved through discussions and mutual agreements. For studies in other languages than English, native researchers confirmed accurate extraction and translation of the data.

### Data synthesis and risk of bias

Given the expected methodological heterogeneity, a narrative synthesis approach was used – a method that primarily relies on the use of text to summarize and explain the synthesis’ findings [24]. This method comprised three consecutive stages: (1) developing a preliminary synthesis, (2) exploring relationships within and between studies, and (3) assessing the robustness of the synthesis.

#### Preliminary synthesis

The preliminary synthesis focused on structuring the included studies to facilitate subsequent analysis, and comprised three sequential steps: grouping and clustering, tabulation, and vote counting. Initially, studies were categorized according to several criteria: relevance to one of the four secondary research questions, the nature of the data (i.e., quantitative or qualitative), the reported outcomes of interest, and the characteristics of the study population (i.e., HCPs or patients). Studies involving HCPs were further grouped by profession, encompassing nurses, doctors, and GPs. Additional subgroups were considered, such as cancer patients and survivors, early-stage and advanced cancer patients, inpatients and outpatients, and fatigued and non-fatigued patients. However, these subgroups were not incorporated in any further analyses due to insufficient data. For the following step, studies were systematically arranged into tables based on the predefined categories. Data on study methods, populations, and outcomes were presented within these tables, thereby enabling comparisons across studies. In the final step of the preliminary synthesis, vote counting was used to quantify the study populations, outcomes, and data types, offering insights into the prevalence of various study characteristics among the included studies.

#### Exploration of relationships within and between studies

The exploration of relationships within and between studies sought to provide a comprehensive overview of the findings relevant to the research questions through a structured four-step process: qualitative case descriptions, idea webbing, analysis of moderator variables and subgroups, and triangulation. All secondary research questions were analyzed separately. For findings related to barriers and facilitators of CRF management, as well as interventions aimed at improving it, the analysis was confined to qualitative case descriptions due to insufficient data for any succeeding analyses.

The qualitative case descriptions were prepared to summarize individual studies and to identify common themes, potential moderator variables, and subgroups. These descriptions strengthened understanding of the data and validated the initial grouping criteria of the preliminary synthesis, as these categories consistently emerged throughout the descriptions. Subsequently, idea webbing was employed to visualize relationships within outcomes. For instance, studies reporting on fatigue screening might address dimensions of the outcome, such as “screening consistency” or “screening in the past month.” Illustrative overviews for all outcomes and their associated dimensions were created. Following, moderator variables and subgroups were evaluated to assess their potential influences on studies’ outcomes. Outcome data for the various subgroups were summarized and compared to identify any potential differences between subgroups and the overall dataset. During this stage, the publication year of studies was considered as a potential moderator. However, the substantial variation within outcomes and the corresponding data fragmentation hindered the identification of any discernible trends in CRF management and guideline adherence over time. Ultimately, triangulation was performed by integrating quantitative and qualitative data through joint displays and narrative weaving. Both data types were visually integrated into the overviews developed through idea webbing to create joint displays, which are provided in Supplementary Materials File 2. Thereafter, narrative weaving was applied, where the quantitative and qualitative findings for each outcome were described together. The concluding phase of triangulation entailed reporting on the “fit” of the data, referring to the coherence between quantitative and qualitative findings, and determining whether the results corroborated, expanded upon, or contradicted each other.

#### Robustness of the synthesis

For the final stage of the narrative synthesis, the robustness of the synthesis was assessed by examining how the quality of the included studies might have influenced the review’s findings. The Mixed Methods Appraisal Tool (MMAT) was used to evaluate the quality of qualitative, randomized controlled trials (RCTs), n-RCTS, quantitative descriptive, and mixed-methods studies through posing five specific questions for each study type [25]. All questions are answered with a low, high, or unclear risk of bias, with the latter indicating that risk of bias cannot be determined with certainty. Prior to evaluating studies’ quality, a calibration session was conducted to standardize the first and second reviewers’ judgements. Both reviewers then independently rated 25% of the included English studies. Disagreements between both reviewers were resolved by finding consensus. If no consensus could be reached, the third and fourth reviewers were consulted. Subsequently, the first reviewer rated the remaining English studies according to the standards established during the calibration session and the discussions among all reviewers. For studies in languages other than English, native researchers rated the studies based upon the corresponding MMAT criteria.

The quality of each study is represented by the number of criteria met, with a single asterisk signifying that only one criterion was satisfied, and five asterisks signifying that all criteria were fulfilled. For mixed-methods studies, there is the premise that the study’s overall quality is constrained by its weakest constituent. Therefore, the overall study quality equals the lowest score among its individual components. For all outcomes considered in the current review, it was examined whether poor study quality—defined as meeting none, one, or two criteria—and the associated biases were linked to certain findings or outcomes, potentially influencing the review’s conclusions.

## Results

### Selection of studie*s*

**Fig. 1:**
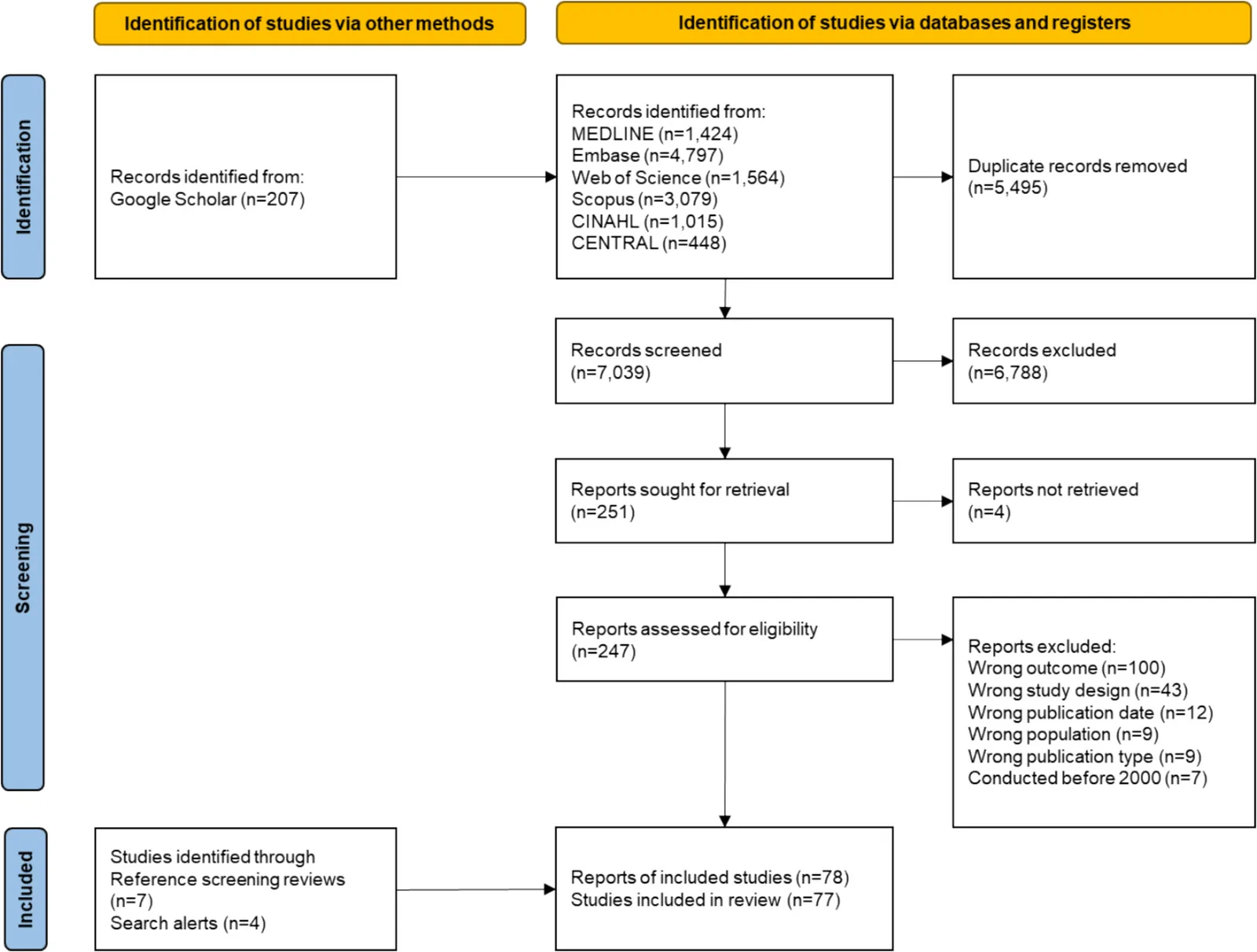
Flow diagram of the literature search and the selection process

The initial electronic database search identified 12,327 records, and an additional 207 records were located through Google Scholar, resulting in 12,534 records altogether. Following duplicate removal, 7039 records were screened based on title and abstract, leaving 251 records for full-text screening, of which sixty-seven reports met the inclusion criteria. Search alerts and reference screening of relevant reviews led to the inclusion of an additional eleven publications, resulting in a total of seventy-seven studies, comprising seventy-eight reports, included in the current review. The search and selection process is visualized in Fig. 1.

### Study characteristics

Of the seventy-seven included studies, fifty-two provided quantitative data, twenty-four qualitative data, and two supplied mixed-methods data. Cancer patients or survivors were included in sixty-six studies, while HCPs were represented in twenty-three studies. In studies with quantitative data, the most frequently reported outcomes were communication (*n* = 23), treatment (*n* = 20) and screening (*n* = 16). In studies with qualitative data, common outcomes included communication (*n* = 19), information (*n* = 13), and both advice and treatment (*n* = 8 each). The main characteristics of the included studies are provided in Supplementary Materials File 3.

### Extent of guideline adherence from the patients’ and the healthcare professionals’ perspective

The results of the current review consider guideline usage as the overarching outcome, offering insights into HCPs’ reported usage of clinical guidelines for fatigue management. Subsequently, outcomes relating to the key stages of the CRF guidelines (i.e., screening, assessment, and treatment), alongside the additional outcomes (i.e., information, communication, advice, and documentation), are discussed in the same sequence as they would generally occur in clinical practice. Accordingly, communication and information about fatigue are addressed first, given that they typically precede fatigue screening and assessment. Succeeding fatigue assessment, HCPs are likely to provide advice, which may be followed by the provision of treatment. Documentation, although discussed last, is integral throughout all phases of fatigue management, beginning with communication and continuing through treatment. It should be noted that, while each outcome is reported separately, they collectively reflect an integrated, nonlinear sequence of steps essential for effective adherence to the CRF guidelines. A summary of the primary outcomes and associated statements from the CRF guidelines is presented in Table 2.

**Table 2:** Summary of key outcomes and corresponding cancer-related fatigue guideline statements

Outcome	Guideline statements	Patient perspective	Healthcare professional perspective
Guideline usage	Fatigue should be screened, assessed, and managed according to clinical practice guidelines^N^	NA	Overall, HCPs’ adherence to CRF guidelines is limited. Organizational procedures for managing CRF are either poorly developed or, in some cases, non-existent
Screening	Screening for fatigue should occur at diagnosis^A,C^ and continue throughout and following treatment^A,C,N^. Screening must be conducted at regular intervals, preferably with validated (semi-)quantitative screening tools, such as the 0–10 NRS^A,C,N^	Fatigue is occasionally screened in patients. However, consistent screening is uncommon, as is the use of validated screening tools. Patients express a preference for incorporating fatigue screening into routine cancer care	Fatigue screening is inconsistently practiced and, when conducted, most HCPs rely on personal observations or informal interviews as screening methods rather than using validated screening instruments
Assessment	Follow-up assessment is recommended for fatigue scores of 3^C^ or 4^A,N^ or higher on the 0–10 NRS. Fatigue assessments should involve the evaluation of fatigue onset, pattern, duration, and interference with daily functioning^A,C,N^. Treatable and contributing factors including comorbidities, medications, and nutrition should be addressed first before continuing with CRF treatment^A,C,N^	In-depth assessment following a positive fatigue screening result is rare, and the methods used to identify the physical causes of fatigue vary considerably. When a clear physical diagnosis is not identified, patients often feel that their experiences of fatigue are being trivialized	Fatigue assessment primarily involves evaluating objective data and related symptoms; however, only a minority of HCPs engage in such practices
Treatment	Non-pharmacologic interventions are preferred for treating CRF, such as (un)supervised physical activity, cognitive behavioral therapy and mindfulness programs. As pharmacological agents show limited efficacy, these should be prescribed with caution^A,C,N^	The discussion and provision of CRF treatment differ widely between practices. Pharmaceuticals are regularly provided to treat CRF, while physical activity is prescribed less frequently, and psychosocial strategies are rarely offered	A variety of CRF treatments are available, with physical activity and psychosocial support frequently cited as treatment recommendations. However, rest and pharmacological interventions remain commonly suggested approaches for treating CRF

The guidelines included in this table are those from the American Society of Clinical Oncology (ASCO) [6], the Canadian Association of Psychosocial Oncology (CAPO) [8], and the National Comprehensive Cancer Network (NCCN) [5]. These three guidelines were the most frequently referenced in the included studies, with the ASCO guideline cited ten times, the CAPO guideline twelve times, and the NCCN guideline twenty-nine times. Each guideline recommendation is linked to its source as follows: ^A^, ASCO; ^C^, CAPO; ^N^, NCCN.

*CRF* cancer-related fatigue, *HCP* healthcare professional, *NA* not applicable, *NRS* numeric rating scale.

#### Guideline usage

Among the four quantitative studies examining the use of guidelines for fatigue management, two revealed a discrepancy between awareness and implementation. Specifically, while 50% and 74% of the HCPs were aware of these guidelines, only 11% and 24% respectively followed them in practice [26, 27]. Additionally, two studies demonstrated that, within the included practices, internal CRF guidelines were either absent or inadequately developed [28, 29]. However, given the limited number of studies reporting on guideline usage and the potential for HCPs to unknowingly follow guidelines based on personal judgement of what constitutes good clinical practice, the findings should be interpreted with caution. Adherence to guidelines for fatigue management could also be reflected in other related outcomes, underscoring the necessity of examining these outcomes for a more comprehensive understanding of guideline adherence.

#### Communication

A total of forty-one studies reported on fatigue communication from patients’ perspectives, and five from HCPs’ perspectives. These studies underscore a variation in fatigue communication practices across healthcare settings and among different HCPs. While HCPs acknowledged they seldom initiated discussions about fatigue, they recognized it as a symptom that was easily and commonly addressed [11, 30–33]. From the patients’ perspective, quantitative data indicated that 34% to 73% of patients discussed fatigue with an HCP at least once [31, 34–43]. Patients noted that fatigue was more frequently addressed during consultations with oncologists compared to those with GPs or nurses [34, 40, 44, 45]. However, during these discussions, patients revealed they regularly encountered dismissive attitudes from HCPs, characterized by disinterest and trivialization of their experiences [32, 41, 46–56]. Such responses fostered frustration and discouraged patients from further disclosing their fatigue, implying a cycle where negative interactions inhibit communication [32].

#### Information

Seventeen studies addressed patients’ experiences with CRF information while six studies focused on HCPs. Although some HCPs attempted to educate patients about CRF, the majority acknowledged that such education was not common [28, 53, 57, 58]. Approximately half of the patients reported not being forewarned about CRF as a side effect or its potential persistence, leaving numerous patients unprepared for the symptom [31, 34, 40, 48, 55–57, 59, 60]. As a result, many described feeling insufficiently informed about fatigue [18, 41, 61, 62]. On a positive note, when patients did receive CRF-related information, it helped to normalize their experiences and alleviate distress, suggesting that adequate information provision may enhance patient outcomes [56, 63].

#### Screening

Fifteen studies discussed fatigue screening from the patients’ perspective, and five considered the HCPs’ perspective. Patients reported experiencing unmet needs, as their desire for routine screening was frequently unaddressed [53, 54, 60]. At the same time, quantitative data indicated that approximately half of patients were asked about the presence of fatigue *at least once*, and 47% to 67% of the patients were consistently screened for fatigue, defined as occurring occasionally or at more than half of the appointments [15, 18, 42, 43, 54, 63–66]. Compared to patients, HCPs showed greater variability in reported screening practices, as 11%, 45%, and 75% of HCPs routinely screened for CRF, of whom only a small minority used validated screening instruments [12, 26, 27, 67]. Rather, HCPs relied on personal observations, informal interviews, or direct questioning as screening methods. They also described that focusing on patients’ daily functioning and presentation during consultations served as a method for fatigue screening [12, 26, 63, 67]. The use of informal screening methods added subjectivity to fatigue screening, a challenge recognized by HCPs as contributing to uncertainty in the screening process [63].

#### Assessment

In total, six studies examined patients’ experiences with fatigue assessment, while five focused on those of HCPs. A small proportion of patients, ranging from 0% to 35%, reported undergoing tests to identify the underlying physical causes of their fatigue [14, 47, 48, 54, 63]. Similarly, 19% and 29% of the HCPs indicated they conducted fatigue assessments, which typically involved investigating related symptoms, such as depression, and gathering objective measures like blood tests and vital signs [12, 53, 63, 68, 69]. For many patients, these tests were perceived as invasive, inconvenient, and trivializing of their fatigue, particularly when they failed to provide a clear physical diagnosis [47, 48, 55].

#### Advice

Overall, twenty studies examined the provision of CRF-related advice from the patients’ perspective and six studies from the perspective of HCPs. Studies among HCPs, when considered collectively, demonstrated a wide variety of advice offered to patients, including recommendations for physical activity, rest, energy management, and education [27, 32, 53, 57, 63, 68]. However, the type of advice provided by individual HCPs was shaped by their personal beliefs, reflecting an absence of standardized practices [53, 63]. As a result, many patients reported receiving limited or ineffective advice, with rest being the most commonly suggested recommendation to alleviate fatigue, despite patients finding it unhelpful [50, 52, 54, 57, 60, 63, 70]. Whether patients received advice also appeared to depend on the presence of fatigue. One study demonstrated that 73% of the fatigued patients received advice on managing fatigue, compared to just 32% of the non-fatigued patients [13]. Another study solely including fatigued patients reported that 72% received advice for fatigue, while in studies conducted among patients with mixed or unreported fatigue levels, the proportion of patients receiving fatigue-related advice ranged from 0 to 44% [14, 31, 35, 64, 71–73]. It should be noted that most studies failed to specify the type of advice provided, leaving the appropriateness of the advice uncertain.

#### Treatment

A total of nineteen studies examined CRF treatment from the patients’ perspective, whereas eleven studies considered HCPs. Collectively, these studies highlight a disparity between HCPs’ proclaimed treatment accessibility and patients’ recollections of being offered these treatments. One study, for instance, found that 66% of HCPs acknowledged the availability of supportive CRF treatments, yet only 30% of their patients recalled being offered treatment [19]. Similarly, in other studies, although 80% of HCP claimed accessible CRF interventions, only 40% and 50% of patients remembered any treatments being offered [17, 39, 42]. Moreover, whereas the majority of HCPs assumed that over 60% of patients were being treated for CRF, just 16% to 61% of patients actually received such care [16, 19, 35–37, 39, 50, 74]. In terms of treatment modalities, patients most frequently described using pharmaceuticals and participating in physical activity for CRF treatment, while psychosocial therapy was rarely utilized [18, 37, 63, 74]. Studies from the HCP perspective revealed a wide range of CRF treatments, including pharmaceuticals, physical activity, and psychosocial therapy, although the variability across studies complicated efforts to identify a dominant treatment approach [11, 12, 31, 39, 53, 58, 63, 67]. A notable discrepancy existed in treatment recommendations between physicians and nurses. Physicians were more likely to recommend pharmaceuticals to treat CRF, with 35% and 70% favoring drug interventions, compared to only 10% and 5% of nurses, respectively [12, 31]. Differences in HCPs’ treatment recommendations not only highlight divergent treatment preferences but also the influence of HCPs in shaping patient care. Another interesting finding was that in one study, 24.6% of the patients who engaged in physical activity and 55.1% of those receiving psychosocial counselling for CRF reported their treatments were recommended or initiated by a physician. In contrast, 83.9% of the patients using pharmaceuticals to treat CRF noted that their treatment was recommended or initiated by an HCP [18]. These findings raise questions about the reported use of non-pharmacological treatments in other studies, and whether these reflect HCPs’ adherence to the CRF guidelines or patients’ independent actions.

#### Documentation

Finally, fifteen studies investigated the documentation of fatigue in clinical records. Unlike other outcomes, where the considered study population might have influenced findings, the objective nature of documentation eliminates these influences. Hence, findings are presented collectively rather than separately for patients and HCPs. Studies demonstrated that several aspects of fatigue management, including information, assessment, and treatment, were rarely documented in patients’ charts [28, 63, 64, 75–82]. Screening was the most frequently documented aspect of fatigue management, with medical charts indicating screening was performed in 23% to 69% of the patients [76, 83, 84]. However, conclusions from these screening practices were rarely noted; the symptom was recorded as present in only 13% to 43% of medical records, and its severity was graded in a mere 3% and 11% [64, 76, 82, 85]. Notably, in studies examining the absence of fatigue, none of the clinical records indicated that a patient was *not* experiencing fatigue [79, 82]. The underreporting of fatigue, its impact, and its management may reflect a failure to properly document care, or indicate broader issues, such as inadequate management of the symptom.

### Barriers and facilitators of healthcare professionals’ guideline adherence

Among four studies exploring HCPs’ barriers to fatigue management, two specifically addressed challenges related to CRF guideline usage. One of these studies highlighted the lack of integration of CRF guidelines into standard care pathways, while the other study pointed out that the guidelines focused more on summarizing evidence than offering actionable steps, leaving HCPs uncertain about how to proceed following assessments [53, 54]. Moreover, three studies identified additional barriers to providing fatigue information, screening, and treatment recommendations, with time constraints affecting all these practices [11, 53, 57]. A lack of knowledge hampered effective screening and treatment of fatigue, and competing priorities limited the ability to offer comprehensive fatigue information and treatment recommendations [11, 53, 57].

Five studies highlighted facilitators to CRF management. Regarding guideline usage, HCPs advocated for more concise and accessible guidelines that were tailored to the specific needs of individual healthcare settings [54]. Other outcomes for which facilitators were reported included fatigue communication, information provision, screening, assessment, and treatment evaluation [11, 26, 54, 58, 63]. Education was seen as essential for enhancing information, assessment, and treatment practices [11, 54, 63]. Moreover, the availability of validated screening tools and their integration into existing workflows were viewed as critical for improving the efficiency of fatigue screening, assessment, and treatment evaluation [54, 63]. Finally, one study underscored the importance of trust, clear communication, and a strong understanding of CRF in fostering more effective CRF communication [58].

### Interventions aimed at enhancing healthcare professionals’ guideline adherence

The examination of intervention studies revealed two distinct approaches: intervention (*n* = 6) and implementation (*n* = 2) studies, which are reported separately due to their methodological divergence. All intervention studies, conducted exclusively in outpatient clinic settings, aimed to enhance symptom management during consultations [30, 77, 79, 80, 84, 86]. Across these studies, HCPs received summaries of patient-reported outcome measures (PROMs) with patients’ self-reported symptom data, either before or during consultations. Notable variability was evident in the visualization of the PROMs, the extent of HCP training in interpretation and addressing the summaries, and the design of the control conditions—ranging from care-as-usual to scenarios where PROMs were completed but not shared with HCPs.

Concerning the four studies aspiring to improve fatigue documentation, two demonstrated increased documentation in the intervention group compared to the control, although only one incorporated statistical analyses and achieved significance [77, 79]. The third study reported no significant improvements in documentation for the intervention group compared to the control group, and the fourth study revealed a mixed outcome: one study site demonstrated a significant increase in documentation under the intervention, while another study site experienced a decrease [80, 84]. At the latter study site, however, baseline documentation in the control group already reached 100%, leaving no room for further improvement. Among the four studies exploring fatigue communication, three failed to identify significant differences between the intervention and control groups [30, 80, 86]. The fourth study did not allow for evaluation of the intervention’s effect on communication due to lack of control data [77].

The two implementation studies sought to promote evidence-based practices for managing CRF within in-patient care, with a particular focus on fatigue information, screening, and assessment [14, 29]. One study followed the National Comprehensive Cancer Network (NCCN) guidelines as evidence-based practice, whereas the other created its own guideline without disclosing the guideline’s content. Both studies targeted nursing staff, assembled project teams, and incorporated structured implementation frameworks. Their strategies involved staff training, designing clinical records for CRF management, and developing educational materials for patients. Both reported substantial improvements in all evaluated aspects of evidence-based practice, indicating that guideline implementation is achievable with sufficient time and resources. Nevertheless, the absence of long-term outcome data creates uncertainty regarding the lasting impact of the implementations.

### Robustness of the synthesis

The potential influence of studies’ quality on this review’s findings was assessed for all measures. While no consistent patterns of influence from study quality were identified, it is important to acknowledge the challenges encountered during this assessment. For instance, consistent reporting issues, particularly in quantitative studies, made it challenging to fully assess study quality. Moreover, the variability in outcome definitions across studies further complicated the evaluation, as the substantial number of sub-outcomes made it difficult to identify trends of influences within outcomes. Despite these challenges, the assessment provided insights into the overall findings, with study quality unlikely to have played a significant role in shaping the current review’s findings. The results of the risk of bias assessment are presented in Table 3.

**Table:** Results of the risk of bias assessment conducted according to the Mixed Methods Appraisal Tool

Studies	Quality criteria																									Quality criteria met
	1.1	1.2	1.3	1.4	1.5	2.1	2.2	2.3	2.4	2.5	3.1	3.2	3.3	3.4	3.5	4.1	4.2	4.3	4.4	4.5	5.1	5.2	5.3	5.4	5.5	
Bradford et al. (2023) [46]	1	1	1	1	1																					*****
Browne et al. (2011) [87]	1	1	1	1	1																					*****
Charles et al. (2021) [47]	1	1	1	1	1																					*****
Corbett et al. (2016) [48]	1	?	1	1	1																					****
Dolgoy et al. (2020) [10]	1	1	1	1	1																					*****
Erickson et al. (2013) [88]	1	1	1	0	?																					***
Fitch et al. (2008) [49]	1	1	1	?	0																					***
Glaus et al. (2002) [50]	1	1	1	1	1																					*****
Horne et al. (2014) [68]	1	1	1	1	1																					*****
John (2010) [70]	1	1	1	1	1																					*****
Krok-Schoen et al. (2019) [32]	1	1	?	1	1																					****
Martin et al. (2020) [11]	1	1	?	1	?																					***
Morgan et al. (2014) [89]	1	0	?	1	0																					**
O'Neill et al. (2023) [52]	1	1	1	1	1																					*****
Pearson et al. (2023) [53]	1	1	1	1	1																					*****
Pertl et al. (2014) [55]	1	1	1	1	0																					****
Piredda et al. (2007) [57]	1	1	?	1	?																					***
Porock and Juenger (2004) [59]	1	1	?	1	1																					****
Robertson and Skär (2021) [58]	1	1	1	0	?																					***
Siegel et al. (2012) [56]	1	?	1	1	1																					****
Synder et al. (2010) [33]	1	1	0	0	1																					***
Spichiger et al. (2012) [60]	1	1	1	1	1																					*****
Tang et al. (2018) [90]	1	1	1	1	1																					*****
Berry et al. (2011) [30]						1	1	0	?	1																***
Nicklasson et al. (2013) [80]						1	1	0	?	1																***
Carpenter et al. (2008) [77]											?	?	0	?	1											*
Carreira et al. (2022) [78]											?	1	1	1	1											****
											1	1	1	1	1											*****
Hilarius et al. (2008) [86]											0	1	0	?	1											**
Leung et al. (2022) [85]											?	1	1	?	1											***
Marino et al. (2020) [79]											1	?	1	?	1											***
Mark et al. (2008) [84]											?	?	1	?	1											**
Milzer et al. (2022) [41]											0	1	0	?	?											*
Tian et al. (2017)^a^ [29]											?	?	1	?	1											**
Wang et al. (2018)^a^ [14]											?	?	1	?	1											**
Al Maqbali et al. (2020) [26]																1	0	1	0	1						***
Alcalde-Castro et al. (2020) [83]																1	1	1	1	1						*****
Blaney et al. (2011) [71]																1	0	1	0	1						***
Bock et al. (2012) [75]																1	?	?	?	1						**
Borneman et al. (2007) [76]																1	?	1	?	1						***
Butt et al. (2008) [61]																1	?	1	?	1						***
Cardoso et al. (2023) [44]																1	?	?	?	?						*
Cheville et al. (2013) [74]																1	1	1	1	1						*****
Chouaïd et al. (2010) [34] and Maloisel et al. (2010) [40]																1	?	1	?	1						***
Collins et al. (2008) [35]																1	?	?	0	1						**
de Ligt et al. (2019) [91]																1	?	1	0	1						***
Díaz et al. (2008) [36]																1	?	1	?	1						***
Dillon and Kelly (2003) [31]																1	?	?	?	?						*
Donovan and Ward (2005 [72])																1	?	1	0	1						***
Harrington et al. (2022) [37]																1	0	1	0	1						***
Hershman et al. (2008) [92]																1	1	?	?	1						***
Hingst et al. (2021) [67]																1	?	?	0	1						**
James et al. (2015) [64]																1	1	1	0	1						****
Jenkins et al. (2013) [73]																1	?	1	0	1						***
La Verde and Arienti (2002) [39]																1	1	?	?	?						**
Luctkar-Flude et al. (2015) [27]																1	0	1	0	1						***
Miller and Kearney (2002) [28]																1	?	?	0	1						**
Nurhesti and Adiputra (2020) [69]																1	?	?	0	?						*
Passik et al. (2002) [42]																1	?	1	?	1						***
Pearson et al. (2015)^a^ [12]																1	0	1	?	1						***
Pearson et al. (2021) [93]																1	?	1	?	1						***
Penalba et al. (2019) [45]																1	?	1	?	1						***
Rau et al. (2020) [16]																1	?	1	?	1						***
Ruiz et al. (2014) [17]																?	?	1	?	1						**
Schmidt et al. (2021) [18]																1	?	?	0	1						**
Schmidt et al. (2022) [62]																1	?	1	?	1						***
Simon et al. (2020) [19]																1	?	?	0	1						**
Smith et al. (2019) [13]																1	?	1	?	1						***
Tarp et al. (2023) [82]																1	?	1	0	1						***
van Ryn et al. (2014) [43]																1	?	1	0	1						***
Walling et al. (2013) [65]																1	?	1	1	1						****
Zucca et al. (2016) [15]																1	0	1	0	1						***
Zucca et al. (2017) [66]																1	?	1	0	1						***
Iguchi et al. (2022) [51]	1	1	?	1	1											?	?	1	?	1	?	?	?	?	0	
Pearson et al. (2017) [54]	1	1	1	1	1											1	0	1	0	1	1	1	1	0	0	***
Schmidt et al. (2016) [81]	1	1	1	0	0											1	?	1	1	1	?	?	1	?	0	*
Tolotti et al. (2021) [63]	1	1	1	1	1											1	?	1	0	1	1	1	1	0	0	***

^a^The study employed both qualitative and quantitative methods and could be considered a mixed-methods study. However, due to the dominance of one method and the lack of triangulation – a core characteristic of mixed-methods research – the study was rated based on its most dominant methodological approach

Study design categories and methodological quality criteria:

1. Qualitative studies; 1.1, Is the qualitative approach appropriate to answer the research question?; 1.2, Are the qualitative data collection methods adequate to address the research question?; 1.3, Are the findings adequately derived from the data?; 1.4, Is the interpretation of results sufficiently substantiated by data?; 1.5, Is there coherence between qualitative data sources, collection, analysis and interpretation?

2. Quantitative randomized controlled trials; 2.1, Is randomization appropriately performed?; 2.2, Are the groups comparable at baseline?; 2.3, Are there complete outcome data?; 2.4, Are outcome assessors blinded to the intervention provided?; 2.5, Did the participants adhere to the assigned intervention?

3. Quantitative non-randomized controlled trials; 3.1, Are the participants representative of the target population?; 3.2, Are measurements appropriate regarding both the outcome and intervention (or exposure)?; 3.3, Are there complete outcome data?; 3.4, Are the confounders accounted for in the design and analysis?; 3.5,

During the study period, is the intervention administered (or exposure occurred) as intended?

4. Quantitative descriptive studies; 4.1, Is the sampling strategy relevant to address the research question?; 4.2, Is the sample representative of the target population?; 4.3, Are the measurements appropriate?; 4.4, Is the risk of nonresponse bias low?; 4.5, Is the statistical analysis appropriate to answer the research question?

5. Mixed-methods studies; 5.1, Is there an adequate rationale for using a mixed methods design to address the research question?; 5.2, Are the different components of the study effectively integrated to answer the research question?; 5.3, Are the outputs of the integration of qualitative and quantitative components adequately interpreted?; 5.4; Are divergences and inconsistencies between quantitative and qualitative results adequately addressed?; 5.5, Do the different components of the study adhere to the quality criteria of each tradition of the methods involved?

Quality rating: 1, high risk of bias; 0, low risk of bias; ?, unclear risk of bias; *, one quality criteria met; *****, five quality criteria met

## Discussion

In summary, the present review highlights suboptimal adherence of HCPs to CRF guidelines. Few studies explicitly explored HCPs’ intentional guideline usage, limiting inferences about deliberate adherence. Nonetheless, evidence reveals suboptimal integration of CRF guidelines into practice. To illustrate, CRF screening varied widely across settings and HCPs indicated to often rely on non-validated screening instruments. Furthermore, HCPs rarely conducted fatigue assessments, and when they did, they tended to focus on underlying physical causes rather than on the fatigue itself. Moreover, there was a discrepancy in treatment practices; while HCPs reported high treatment availability, patients often claimed these interventions were not offered to them. Concerning treatment methods, pharmaceuticals remained a common approach, although physical activity was regularly mentioned as an alternative. Inadequate CRF management was attributed to barriers among HCPs, such as time constraints and lack of knowledge. Finally, intervention studies showed minimal success in improving fatigue management in clinical practice, whereas implementation studies demonstrated more favorable outcomes, albeit at the cost of significant resource investment.

To understand the potential changes in CRF management since the clinical guidelines’ publication, comparing this review’s findings with those from the 2004 review offers valuable insight [9]. Notably, none of the studies in the previous review addressed fatigue screening, thereby precluding direct alignment on this outcome. Two studies from the prior review focused on CRF assessment, revealing that HCPs primarily prioritized identifying underlying physical causes of fatigue rather than exploring the fatigue itself. Similarly, the current review indicates that the multidimensional nature of fatigue still receives insufficient attention. In contrast, treatment strategies have progressed over time, with a marked increase in the proportion of patients receiving CRF treatment. Although pharmaceuticals remain a common treatment option, as highlighted in both reviews, there is a noticeable transition from the earlier emphasis on rest to the current promoting of physical activity. Moreover, psychosocial therapies are more frequently reported compared to earlier practices, although their adoption remains limited. The comparison between the two reviews underscores both improvements and ongoing gaps in CRF management. While treatment approaches have advanced, uncertainties persist regarding whether these developments result from continued refinement of clinical guidelines, a broader shift in the clinical perception of CRF, or the interplay between these factors.

The present review identified numerous barriers HCPs encounter in managing CRF, including time constraints, knowledge gaps, and competing priorities. Additional barriers, such as inadequate referral pathways and undefined responsibilities in CRF management for HCP, were reported in studies involving clinical care teams and allied HCPs [12, 53, 54, 94]. These barriers to CRF management are likely rooted in broader organizational and policy-related insufficiencies. For instance, competing priorities may arise from restricted consultation times, forcing HCPs to prioritize certain aspects of care. Similarly, limited knowledge could indicate shortcomings in HCP training and education, while unclear roles may be attributed to poorly defined policies and communication strategies. These barriers are also likely to contribute to patients’ barriers in CRF management. Ambiguities in the roles of HCP can create confusion for patients regarding whom to approach for fatigue [95]. Moreover, conflicting priorities, lack of knowledge, and role uncertainty may influence the information and education patients receive, leading them to believe fatigue is inevitable, not prioritized, or unworthy of mentioning [42, 96, 97]. One study lends support to the interdependence of barriers, describing how limited consultation times prompted HCPs to deprioritize CRF conversations, which in turn discouraged patients from raising the topic in subsequent visits [94]. These interconnected barriers underscore the importance of addressing systemic shortcomings to improve both HCPs’ adherence to CRF guidelines and the quality of patient care.

One might wonder whether the clinical management of CRF and that of other common cancer-related symptoms show similarities. Numerous studies included in the present review assessed both fatigue and either pain or distress management, with shared methodologies, settings, and populations enabling direct comparison of how these symptoms are managed. These studies suggest that, in contrast to fatigue, HCPs are more inclined to discuss, screen, treat, and document pain, while they are less likely to address distress during consultations, even when controlling for symptom prevalence [13, 15, 19, 30, 43, 61, 66, 79, 83, 86, 91]. One possible explanation for the imbalance in symptom management may lie in the differences in symptom perceptions between HCPs and patients. Research indicates that HCPs consistently underestimate the severity of fatigue and distress and believe that pain had a greater burden on patients’ lives than fatigue, in contrast to patients, who perceive fatigue as more debilitating than pain [98–101]. Such misconceptions about the burden of fatigue and distress are likely to influence the focus of consultations. Medical issues like pain are often prioritized by HCPs, who tend to neglect or minimally discuss the concerns of fatigue and distress [30, 102, 103]. Additionally, as HCPs generally dominate the speaking time during consultations, they foster an environment in which their clinical priorities took precedence [102]. Accordingly, the prevailing consultation dynamics and HCPs’ misunderstanding concerning the impact of fatigue may not only explain why fatigue is less frequently addressed than pain, but also why patients often feel that their fatigue is being dismissed or invalidated by HCPs, as highlighted in the current review. Although a systematic analysis of the other symptoms was not conducted, the findings emphasize the need to enhance HCPs’ understanding of CRF and to balance the focus of consultations, both of which are essential for improving CRF management.

An insightful finding from this review is that patients appear more likely to address fatigue with oncologists than with nurses or GPs. This may stem from patients’ perception that fatigue is a medical issue tied to cancer treatment [48, 56, 90, 104]. Research indicates that patients prefer addressing treatment-related concerns with oncologists, who are viewed as specialists. In contrast, nurses and GPs are typically seen as more involved in general health management and perceived as lacking specific expertise in treatment-related concerns [105–107]. Such distinction may explain patients’ preferences for oncologists when discussing CRF. Furthermore, the medical-oriented view of fatigue may inadvertently lead patients to assume it is an unavoidable side effect of treatment with no possible relief, thus discouraging them from raising the issue. Indeed, studies highlight that patients do not mention or seek treatment for CRF as they believe it is an inherent byproduct of cancer treatment [42, 97]. However, CRF is a multifaceted symptom, driven by both the effects of cancer and its treatment, as well as psychosocial influences [108]. Therefore, the medical framing of CRF potentially limits patients’ self-management and access to interventions. Accordingly, the multidimensional nature of CRF should be introduced early in the treatment trajectory, encouraging patients to disclose symptoms more openly to different HCPs.

### Reflection upon the synthesis process

The narrative synthesis process is inherently subjective, with decisions on the approaches taken in both the preliminary and main analyses dependent on the researchers’ judgments. To mitigate the influence of subjectivity, regular discussions among the research team and consistent documentation were maintained throughout the process. Additionally, the maps created through idea webbing are included in the supplementary materials to promote transparency. In terms of triangulation, while overall confirmation between qualitative and quantitative results was observed, some contradictions surfaced, particularly in fatigue screening practices. Qualitative studies reported poorer screening practices compared to quantitative research. However, these discrepancies may reflect the nature of qualitative research, which may highlight more intense or negative experiences that are less likely to be captured in quantitative studies [109]. Moreover, the vast variability in screening practices within both types of research raises the possibility that apparent inconsistencies between quantitative and qualitative studies may reflect actual variations in practice. For other outcomes, qualitative and quantitative data complement one another. To illustrate, whereas quantitative data indicated whether patients received information about fatigue, qualitative findings delved into its significance and impact on patients. Likewise, qualitative studies revealed that, despite conversations about fatigue, patients often felt misunderstood or dismissed. Ultimately, the narrative synthesis method provided a flexible method for integrating diverse findings, with the integration of quantitative and qualitative results facilitating a comprehensive understanding of CRF management.

### Limitations of the literature

The reviewed literature revealed several limitations that complicate drawing reliable conclusions about CRF management and HCPs’ adherence to clinical guidelines. A central issue was the lack of standardized definitions for CRF management. For instance, the term “fatigue assessment” was occasionally misused as a synonym for fatigue screening. Moreover, numerous studies failed to specify which aspects of CRF management (i.e., screening, assessment, treatment) were addressed when reporting on “fatigue communication.” Consequently, participants may have varying interpretations of the term, associating it with different components of CRF management. Similarly, terms for CRF treatment such as “physical activity” or “psychosocial counseling” were often undefined, encompassing a range of interventions from single home-based activities to complex, multi-faceted programs. Furthermore, it remains unclear whether the reported CRF treatments were initiated by HCPs, in accordance with the guidelines, or independently by patients. Together, these irregularities introduce significant variation in outcome measures, questioning whether observed differences between studies reflect genuine disparities in practice, or rather the inconsistent use of measures and definitions. Additionally, discrepancies between clinical records and patient-reported experiences on CRF management raise doubts on the reliability of employing medical records as primary data sources. The limitations collectively underline the challenges involved in accurately measuring and interpreting CRF management practices.

### Strengths and limitations of the review

One of the main strengths of the review is the inclusion of non-English studies, offering a more inclusive perspective on CRF management. The incorporation of both qualitative and quantitative findings further strengthens the review, with the narrative synthesis approach enhancing the cohesive integration of both types of evidence. Additionally, the inclusion of six different databases, along with Google Scholar, significantly expanded the reach and thoroughness of the search. However, despite efforts to capture all relevant studies, some may have been overlooked due to limitations in the search strings, particularly studies that mention fatigue in the full text but not the abstract. Expanding the search string by excluding the fatigue term would have led to an unmanageable number of records. Therefore, to mitigate the issue, the search was supplemented with reference searching from systematic reviews and the use of Google Scholar for full-text searches.

### Implications for future research

To advance CRF management and to enhance HCPs’ adherence to guidelines, future research may prioritize several critical areas. One key priority is ensuring the consistent use of standardized terminology for CRF management, such as screening, assessment, and treatment. These measures are already embedded in CRF guidelines, and adopting them across studies will increase the reliability, validity, and applicability of research findings. Moreover, it is important to understand why certain interventions have had limited success in improving CRF management, even when they incorporate facilitators identified by HCPs. Qualitative follow-up research could uncover reasons for unsuccessful interventions and identify crucial intervention components to enhance intervention feasibility and effectiveness. Additionally, research should examine how healthcare system structures, such as staffing and workflows, influence HCPs’ experienced barriers to CRF management. It is argued that barriers at the HCPs’ level are fundamentally systemic in nature and addressing them within interventions will increase interventions’ effectiveness and long-term sustainability. Simultaneously, future research should adopt a more comprehensive approach to identifying the barriers experienced by HCPs. Given that existing studies on HCPs’ barriers often rely on open-ended questions or focus on a limited subset of barriers, the complete range of barriers to CRF management may not yet be fully explored. Lastly, patient preferences for CRF management could be explored. Although research indicates that guidelines are more likely to be implemented when they consider patient needs and preferences, these have not been considered in the development of the CRF guidelines [110]. Therefore, examining patient preferences is a valuable step toward promoting CRF guideline implementation and adherence.

### Implications for clinical practice

Improving CRF management requires advancements in clinical practice. Standardized screening and assessment protocols should be embedded into institutional policies, with staff trained to use them during routine visits. Incorporating these protocols into workflows and electronic health records can contribute to consistent and reliable CRF care. Additionally, enhancing HCP education is essential for improving the recognition and treatment of CRF. This education must cover a broad range of therapeutic strategies, reducing reliance on pharmaceuticals and promoting evidence-based non-pharmacological interventions. Well-educated HCPs can, in turn, inform patients about the multidimensional nature of CRF, reframing it as a controllable symptom rather than an unmanageable consequence of cancer treatment. Encouraging patients to discuss fatigue with various HCPs, rather than solely relying on oncologists, is crucial for facilitating timely and comprehensive support. Finally, systemic changes within healthcare organizations are essential to overcome the barriers HCPs face in managing CRF. Advocating for structural reforms will enhance CRF care across clinical settings.

## Supplementary Information

Below is the link to the electronic supplementary material.

ESM 1 (145 KB PDF)

ESM 2 (476 KB PDF)

ESM 3 (314 KB PDF)

ESM 4 (195 KB PDF)

## Data Availability

The results of the main analysis upon which the manuscript is written are published in online supplementary materials. Any other form of data compiled and processed during the current study are available upon reasonable request.
