# Supplementary material for: The gap between guidelines and clinical practice in cancer-related fatigue management: a mixed-methods systematic review
Source: J Cancer Surviv. 2025 Jul 18;20(Suppl 1):1220–39. doi: 10.1007/s11764-025-01866-6 (PMC13676525; doi:10.1007/s11764-025-01866-6)
Supplement: Supplementary file 1 [file 11764_2025_1866_MOESM1_ESM.pdf]

Supplementary Materials: File 1

The Gap Between Guidelines and Clinical Practice in Cancer-Related Fatigue Management: A Mixed-Methods Systematic Review

Journal of Cancer Survivorship

Marijn Witziers[a], Anne Looijmans[a], Ning Jia[a], & Mariët Hagedoorn[a]

[a], Department of Health Psychology, University Medical Centre Groningen, University of Groningen, the Netherlands

Correspondence: Marijn Witziers

Email: m.witziers@umcg.nl

Table 1. Search strategy MEDLINE database.

#1	("health personnel"[MESH] OR "delivery of health care"[MeSH] OR “attitude to health”[MeSH] OR “attitude of health personnel”[MESH] OR "health personnel"[tiab] OR "health provider*"[tiab] OR "health care provider*"[tiab] OR "healthcare provider*"[tiab] OR "healthcare worker*"[tiab] OR "health care worker*"[tiab] OR "healthcare professional*"[tiab] OR "health care professional*"[tiab] OR “health professional*”[tiab] OR "medical specialist*"[tiab] OR oncologist*[tiab] OR nurse*[tiab] OR physician*[tiab] OR practitioner*[tiab] OR clinician*[tiab] OR surgeon*[tiab] OR hematologist*[tiab] OR staff[tiab] OR personnel[tiab])
#2	("guideline adherence"[MESH] OR (guideline*[tiab] AND (adher*[tiab] OR complian*[tiab])) OR ((“cancer-related fatigue”[tiab] OR CRF[tiab]) AND (“health services needs and demand”[MeSH] OR “unmet need”[tiab:~3] OR “unmet needs”[tiab:~3])))
#3	((neoplasms[MeSH] OR "cancer patient*"[tiab] OR "cancer survivor*"[tiab] OR "cancer population*"[tiab]) AND (fatigue[MeSH] OR fatigue*[tiab] OR tired*[tiab] OR exhaust*[tiab]))
#4	(“cancer-related fatigue”[tiab] OR “cancer fatigue”[tiab:~3] OR “cancer fatigued”[tiab:~3])
#5	(“guidelines as topic”[MeSH] OR “guideline adherence”[MeSH] OR guideline*[tiab] OR “evidence-based healthcare”[tiab] OR “evidence-based health care”[tiab] OR “evidence-based practice*”[tiab] OR “current practice*”[tiab] OR “current management”[tiab] OR “clinical practice*”[tiab] OR “reported practice*”[tiab])
#6	(“fatigue screening”[tiab:~3] OR “fatigue screen”[tiab:~3] OR “fatigue screened”[tiab:~3] OR “fatigue assessment”[tiab:~3] OR “fatigue assess”[tiab:~3] OR “fatigue assessed”[tiab:~3] OR “fatigue assessing”[tiab:~3] OR “fatigue management”[tiab:~3] OR “fatigue manage”[tiab:~3] OR “fatigue managed”[tiab:~3] OR “managing fatigue”[tiab:~3] OR “CRF screening”[tiab:~3] OR “CRF screen”[tiab:~3] OR “CRF screened”[tiab:~3] OR “CRF assessment”[tiab:~3] OR “CRF assess”[tiab:~3] OR “CRF assessed”[tiab:~3] OR “CRF assessing”[tiab:~3] OR “CRF management”[tiab:~3] OR “CRF manage”[tiab:~3] OR “CRF managed”[tiab:~3] OR “managing CRF”[tiab:~3] OR “cancer-related fatigue screening”[tiab:~3] OR “cancer-related fatigue screen”[tiab:~3] OR “cancer-related fatigue screened”[tiab:~3] OR “cancer-related fatigue assessment”[tiab:~3] OR “cancer-related fatigue assess”[tiab:~3] OR “cancer-related fatigue assessed”[tiab:~3] OR “cancer-related fatigue assessing”[tiab:~3] OR “cancer-related fatigue management”[tiab:~3] OR “cancer-related fatigue manage”[tiab:~3] OR “cancer-related fatigue managed”[tiab:~3] OR “managing cancer-related fatigue”[tiab:~3])

#7	("patient perspective"[tiab:~3] OR "patients perspective"[tiab:~3] OR "patient perspectives"[tiab:~3] OR "patients perspectives"[tiab:~3] OR "patient experience"[tiab:~3] OR "patients experience"[tiab:~3] OR "patient experiences"[tiab:~3] OR "patients experiences"[tiab:~3] OR "patient perception"[tiab:~3] OR "patients perception"[tiab:~3] OR "patient perceptions"[tiab:~3] OR "patients perceptions"[tiab:~3] OR "patient view"[tiab:~3] OR "patients view"[tiab:~3] OR "fatigue experience"[tiab:~3] OR "fatigue experiences"[tiab:~3] OR "impact fatigue"[tiab:~2])
#8	(#1 OR #2) AND (#3 OR #4) AND (#5 OR #6 OR #7)

Table 2. Search strategy Embase database.

#1	(‘health care personnel’/exp OR ‘health care delivery’/exp OR ‘attitude to health’/exp OR ‘health personnel attitude’/exp OR ‘health personnel’:ab,ti,kw OR ‘health provider*’:ab,ti,kw OR ‘health care provider*’:ab,ti,kw OR ‘healthcare provider*’:ab,ti,kw OR ‘healthcare worker*’:ab,ti,kw OR ‘health care worker*’:ab,ti,kw OR ‘healthcare professional*’:ab,ti,kw OR ‘health care professional*’:ab,ti,kw OR ‘health professional*’:ab,ti,kw OR ‘medical specialist*’:ab,ti,kw OR oncologist*:ab,ti,kw OR nurse*:ab,ti,kw OR physician*:ab,ti,kw OR practitioner*:ab,ti,kw OR clinician*:ab,ti,kw OR surgeon*:ab,ti,kw OR hematologist*:ab,ti,kw OR staff:ab,ti,kw OR personnel:ab,ti,kw)
#2	(‘protocol compliance’/exp OR (guideline*:ab,ti,kw AND (adher*:ab,ti,kw OR complian*:ab,ti,kw)) OR ((‘cancer-related fatigue’:ab,ti,kw OR CRF:ab,ti,kw) AND (‘health care need’/exp OR (unmet NEAR/3 need*):ab,ti)))
#3	((neoplasms/exp OR ‘cancer patient*’:ab,ti,kw OR ‘cancer survivor*’:ab,ti,kw OR ‘cancer population*’:ab,ti,kw) AND (fatigue/exp OR fatigue*:ab,ti,kw OR tired*:ab,ti,kw OR exhaust*:ab,ti,kw))
#4	(‘cancer-related fatigue’:ab,ti,kw OR (cancer NEAR/3 fatigue*):ab,ti)
#5	(‘pratice guideline’/exp OR ‘protocol compliance’/exp OR guideline*:ab,ti,kw OR ‘evidence based healthcare’:ab,ti,kw OR ‘evidence based health care’:ab,ti,kw OR ‘evidence-based practice*’:ab,ti,kw OR ‘current practice*’:ab,ti,kw OR ‘current management’:ab,ti,kw OR ‘clinical practice*’:ab,ti,kw OR ‘reported practice*’:ab,ti,kw)
#6	((fatigue NEAR/3 screen*):ab,ti OR (fatigue NEAR/3 assess*):ab,ti OR (fatigue NEAR/3 manag*):ab,ti OR (CRF NEAR/3 screen*):ab,ti OR (CRF NEAR/3 assess*):ab,ti OR (CRF NEAR/3 manag*):ab,ti OR (‘cancer-related fatigue’ NEAR/3 screen*):ab,ti OR (‘cancer-related fatigue’ NEAR/3 assess*):ab,ti OR (‘cancer-related fatigue’ NEAR/3 manag*):ab,ti)
#7	((patient* NEAR/3 perspective*):ab,ti OR (patient* NEAR/3 experience*):ab,ti OR (patient* NEAR/3 perception*):ab,ti OR (patient* NEAR/3 view):ab,ti OR (fatigue NEAR/3 experience*):ab,ti OR (impact NEAR/2 fatigue):ab,ti)
#8	(#1 OR #2) AND (#3 OR #4) AND (#5 OR #6 OR #7)

Table 3. Search strategy Web of Science database.

#1	TS=("health personnel" OR (attitude* NEAR/3 health*) OR "health provider*" OR "health care provider*" OR "healthcare provider*" OR "healthcare worker*" OR "health care worker*" OR "healthcare professional*" OR "health care professional*" OR "health professional*" OR "medical specialist*" OR oncologist* OR nurse* OR physician* OR practitioner* OR clinician* OR surgeon* OR hematologist* OR staff OR personnel)
#2	TS=("guideline adherence" OR (guideline* AND (adher* OR complian*)) OR (("cancer-related fatigue" OR CRF) AND (unmet NEAR/3 need*)))
#3	TS=((neoplasm* OR cancer*) AND (fatigue* OR tired* OR exhaust*))
#4	TS=("cancer-related fatigue" OR (cancer NEAR/3 fatigue))
#5	TS=(guideline* OR "evidence-based healthcare" OR "evidence-based health care" OR "evidence-based practice*" OR "current practice*" OR "current management" OR "clinical practice*" OR "reported practice*")
#6	TS=((fatigue NEAR/3 screen*) OR (fatigue NEAR/3 assess*) OR (fatigue NEAR/3 manag*) OR (CRF NEAR/3 screen*) OR (CRF NEAR/3 assess*) OR (CRF NEAR/3 manag*) OR ("cancer-related fatigue" NEAR/3 screen*) OR ("cancer-related fatigue" NEAR/3 assess*) OR ("cancer-related fatigue" NEAR/3 manag*))
#7	TS=((patient* NEAR/3 perspective*) OR (patient* NEAR/3 experience*) OR (patient* NEAR/3 perception*) OR (patient* NEAR/3 view) OR (fatigue NEAR/3 experience*) OR (fatigue NEAR/2 impact))
#8	(#1 OR #2) AND (#3 OR #4) AND (#5 OR #6 OR #7)

Table 4. Search strategy Scopus database.

#1	("health personnel" OR (attitude* W/3 health*) OR "health provider*" OR "health care provider*" OR "healthcare provider*" OR "healthcare worker*" OR "health care worker*" OR "healthcare professional*" OR "health care professional*" OR "health professional*" OR "medical specialist*" OR oncologist* OR nurse* OR physician* OR practitioner* OR clinician* OR surgeon* OR hematologist* OR staff OR personnel)
#2	(“guideline adherence” OR (guideline* AND (adher* OR complian*)) OR ((“cancer-related fatigue” OR CRF) AND (unmet W/3 need*)))
#3	((neoplasm* OR cancer*) AND (fatigue* OR tired* OR exhaust*))
#4	(“cancer-related fatigue” OR (cancer W/3 fatigue))
#5	(guideline* OR “evidence-based healthcare” OR “evidence-based health care” OR “evidence-based practice*” OR “current practice*” OR “current management” OR “clinical practice*” OR “reported practice*”)
#6	((fatigue W/3 screen*) OR (fatigue W/3 assess*) OR (fatigue W/3 manag*) OR (CRF W/3 screen*) OR (CRF W/3 assess*) OR (CRF W/3 manag*) OR (“cancer-related fatigue” W/3 screen*) OR (“cancer-related fatigue” W/3 assess*) OR (“cancer-related fatigue” W/3 manag*))
#7	((patient* W/3 perspective*) OR (patient* W/3 experience*) OR (patient* W/3 perception*) OR (patient* W/3 view) OR (fatigue W/3 experience*) OR (fatigue W/2 impact))
#8	(#1 OR #2) AND (#3 OR #4) AND (#5 OR #6 OR #7)

Table 5. Search strategy CINAHL database.

#1	(MH “health personnel+” OR MH “health care delivery+ “ OR MH “attitude to health+” OR MH “attitude of health personnel+” OR TI "health personnel" OR AB "health personnel" OR TI "health provider*" OR AB "health provider*" OR TI "health care provider*" OR AB "health care provider*" OR TI "healthcare provider*" OR AB "healthcare provider*" OR TI "healthcare worker*" OR AB "healthcare worker*" OR TI "health care worker*" OR AB "health care worker*" OR TI "healthcare professional*" OR AB "healthcare professional*" OR TI "health care professional*" OR AB "health care professional*" OR TI "health professional*" OR AB "health professional*" OR TI "medical specialist*" OR AB "medical specialist*" OR TI oncologist* OR AB oncologist* OR TI nurse* OR AB nurse* OR TI physician* OR AB physician* OR TI practitioner* OR AB practitioner* OR TI clinician* OR AB clinician* OR TI surgeon* OR AB surgeon* OR TI hematologist* OR AB hematologist* OR TI staff OR AB staff OR TI personnel OR AB personnel)
#2	(MH “guideline adherence+” OR ((TI guideline* OR AB guideline*) AND (TI adher* OR AB adher* OR TI complian* OR AB complian*)) OR (TI “cancer-related fatigue” OR AB “cancer-related fatigue” OR TI CRF OR AB CRF) AND (MH “health services needs and demand+” OR TI (unmet N3 need*) OR AB (unmet N3 need*)))
#3	((MH “neoplasms+” OR TI “cancer patient*” OR AB “cancer patient*” OR TI “cancer survivor*” OR AB “cancer survivor*” OR TI “cancer population*” OR AB “cancer population*”) AND (MH “fatigue+” OR TI fatigue* OR AB fatigue* OR TI tired* OR AB tired* OR TI exhaust* OR AB exhaust*))
#4	(TI “cancer-related fatigue” OR AB “cancer-related fatigue” OR TI (cancer N3 fatigue*) OR AB (cancer N3 fatigue*))
#5	(MH “practice guidelines+” OR MH “guideline adherence+” OR TI guideline* OR TI “evidence-based healthcare” OR TI “evidence-based health care” OR TI “evidence-based practice*” OR TI “current practice*” OR TI “current management” OR TI “clinical practice*” OR TI “reported practice*” OR AB guideline* OR AB “evidence-based healthcare” OR AB “evidence-based health care” OR AB “evidence-based practice*” OR AB “current practice*” OR AB “current management” OR AB “clinical practice*” OR AB “reported practice*”)
#6	(TI (fatigue N3 screen*) OR TI (fatigue N3 assess*) OR TI (fatigue manag*) OR AB (fatigue N3 screen*) OR AB (fatigue N3 assess*) OR AB (fatigue manag*) OR TI (CRF N3 screen*) OR TI (CRF N3 assess*) OR TI (CRF N3 manag*) OR AB (CRF N3 screen*) OR AB (CRF N3 assess*) OR AB (CRF N3 manag*) OR TI (“cancer-related fatigue” N3 screen*) OR TI (“cancer-related fatigue” N3 assess*) OR TI (“cancer-related

	fatigue” N3 manag*) OR AB (“cancer-related fatigue” N3 screen*) OR AB (“cancer-related fatigue” N3 assess*) OR AB (“cancer-related fatigue” N3 manag*))
#7	(TI (patient* N3 perspective*) OR TI (patient* N3 experience*) OR TI (patient* N3 perception*) OR TI (patient* N3 view) OR TI (fatigue N3 experience*) OR TI (fatigue N2 impact)) OR AB (patient* N3 perspective*) OR AB (patient* N3 experience*) OR AB (patient* N3 perception*) OR AB (patient* N3 view) OR AB (fatigue N3 experience*) OR AB (fatigue N2 impact))
#8	(#1 OR #2) AND (#3 OR #4) AND (#5 OR #6 OR #7)

Table 6. Search strategy CENTRAL database.

#1	(“health personnel” OR (attitude NEAR/3 health*) OR (health NEXT provider*) OR (“health care” NEXT provider*) OR (healthcare NEXT provider*) OR (healthcare NEXT worker*) OR (“health care” NEXT worker*) OR (healthcare NEXT professional*) OR (“health care” NEXT professional*) OR (health NEXT professional*) OR (medical NEXT specialist*) OR oncologist* OR nurse* OR physician* OR practitioner* OR clinician* OR surgeon* OR hematologist* OR staff OR personnel)
#2	((guideline* AND (adher* OR complian*)) OR ((“cancer-related fatigue” OR CRF) AND (unmet NEAR/3 need*)))
#3	((neoplasm* OR cancer*) AND (fatigue* OR tired* OR exhaust*))
#4	(“cancer-related fatigue” OR (cancer NEAR/3 fatigue))
#5	(guideline* OR “evidence-based healthcare” OR “evidence based health care” OR (“evidence-based” NEXT practice*) OR (current NEXT practice*) OR “current management” OR (clinical NEXT practice*) OR (reported NEXT practice*))
#6	((fatigue NEXT screen*) OR (fatigue NEXT assess*) OR (fatigue NEXT manag*) OR (CRF NEXT screen*) OR (CRF NEXT assess*) OR (CRF NEXT manag*) OR (“cancer-related fatigue” NEXT screen*) OR (“cancer-related fatigue” NEXT assess*) OR (“cancer-related fatigue” NEXT manag*))
#7	((patient* NEAR/3 perspective*) OR (patient* NEAR/3 experience*) OR (patient* NEAR/3 perception*) OR (patient* NEAR/3 view) OR (fatigue NEAR/3 experience*) OR (impact NEAR/2 fatigue))
#8	(#1 OR #2) AND (#3 OR #4) AND (#5 OR #6 OR #7)
