# Supplementary material for: The gap between guidelines and clinical practice in cancer-related fatigue management: a mixed-methods systematic review
Source: J Cancer Surviv. 2025 Jul 18;20(Suppl 1):1220–39. doi: 10.1007/s11764-025-01866-6 (PMC13676525; doi:10.1007/s11764-025-01866-6)
Supplement: Supplementary file 2 [file 11764_2025_1866_MOESM2_ESM.pdf]

Supplementary Materials: File 2

The Gap Between Guidelines and Clinical Practice in Cancer-Related Fatigue Management: A Mixed-Methods Systematic Review

Journal of Cancer Survivorship

Marijn Witziers[a]*, Anne Looijmans*[a]*, Ning Jia*[a]*, & Mariët Hagedoorn*[a]

[a],Department of Health Psychology, University Medical Centre Groningen, University of Groningen, the Netherlands

Correspondence: Marijn Witziers

Email: m.witziers@umcg.nl

Healthcare professionals - Guideline usage

Fatigue Guidelines

Screening

Fatigue screening guidelines (breast cancer survivors guideline)<br>1. Awareness<br>2. Implements routinely	1. Yes: 46.3%<br>2. Yes: 11%	Luctkar-Flude (2015)

Advice/treatment

Physical activity guidelines (breast cancer survivors guideline)<br>1. Awareness<br>2. Implements routinely	1. Yes: 68.3%<br>2. Yes: 37.8%	Luctkar-Flude (2015)

Assessment

Guidelines usage for CRF assessment	Yes: 23.9%	Al Maqbali (2020)

Internal guidelines/procedures

Indicates poor scores related to these 2 questions:<br>1. If your system uses clinical pathways, do they incorporate fatigue assessment, interventions and outcomes?<br>2. Is there a written standard of practice that articulates the method and frequency for documenting fatigue assessment?	Miller (2002)
No nursing procedures or records sheets existed regarding CRF	Tian (2017)

Figure 1. Idea webbing and joint display for the healthcare professionals' perspective on the outcome of guideline usage.

Note: Green shading represents quantitative data.

CRF, cancer-related fatigue.

Healthcare professionals - Communication

Fatigue communication

Ease

Clinicians felt that fatigue, of all symptoms, is most easily communicated.	Krok-Schoen (2019)

Discussion during visits

Discusses fatigue during patient visits	Yes: ~13 (~87%)	Snyder (2010)

Initiated by whom?

1. Discussion initiated by clinician<br>2. Discussion initiated by patient/family	1. 93 (31.5%)<br>2. 112 (38.0%)	Berry (2011)
1. Fatigue mentioned by patient on most visits<br>2. Fatigue mentioned by patient on every visit	1. Yes for both nurses and physicians.<br>2. Nurses reported twice as many patients report fatigue every visit compared to physicians	Dillon (2003)
Address CRF spontaneously during consultation	No: 12 (60%)	Martin (2020)

Figure 2a. Idea webbing and joint display for the healthcare professionals' perspective on the outcome of communication.

Note: Quantitative data is represented by green shading, while qualitative data is represented by blue shading.

Patients - Communication

Fatigue communication

Initiated by

Discussion initiated by	- 31.5% clinician<br>- 38% patient/family	Berry (2011)

Patients reports when fatigued

Although normalizing fatigue as a side effect relieved participants' concern, it might have undermined their efforts to address it. Indicatively, a few participants who identified treatment as the sole cause of their fatigue did not even report it to their providers because they perceived it as untreatable as well as inevitable.		Siegel (2012)
- "Participants in this study, for the most part, did not talk with health care professionals about the fatigue they were experiencing."<br>- "Most did not raise the topic with their care providers, nor did the health care professionals raise it with them, as far as they could recall."		Fitch (2008)
Patients did express frustration in the clinicians' responses to their complaints about fatigue. This led patients to reduce efforts to communicate their fatigue. One patient noted, "I'm tired, but unfortunately that's not important to them." Another said, "…I would tell him my symptom but eventually they're going to say, 'It's because of the medicine,' nothing else… why tell them in the first place?"		Krok-Schoen (2019)
If fatigue experienced, discuss this with	- 77% oncologists<br>- 34% GP<br>- 33% nurse	Cardoso (2023)
Actively reported suffering from fatigue	Yes: 56.2%	Rau (2020)
Did you inform the nurses who were caring for you about your fatigue?	- Never: 30%<br>- At least once: 23.5%<br>- Several times: 47%	Tolotti (2021)

General communication

Half of participants spontaneously mentioned that they have raised the problem with a health care provider. Among those, only two participants had been informed about the benefits of exercise, and in one case, the participant was already physically active.		Charles (2021)
13 patients (52%) has never discussed CRF or fatigue with their healthcare team		Dolgoy (2020)
20% of the patients has had a discussion about fatigue (with a nurse, physician, or nutritionist)		Erickson (2013)
- "Participants in this study, for the most part, did not talk with health care professionals about the fatigue they were experiencing."<br>- "Most did not raise the topic with their care providers, nor did the health care professionals raise it with them, as far as they could recall."		Fitch (2008)
5 patients (of 8; 62.5%) has discussed symptoms of the burden of their symptoms in regard to fatigue		Iguchi (2022)
Approximately 36% of the patients discusses fatigue during visits.		Snyder (2012)
Few patients said that fatigue had not been mentioned or that they could not remember whether this topic had been discussed.		Spichiger (2012)
Mentioning or discussing fatigue by the doctors was greatly missed.		Glaus (2002)
Discussed fatigue	Yes: 72.9%	Diaz (2008)
Discussed fatigue	No: more than 15%	Dillon (2003)
Who first brought up the topic of fatigue?	You: 76%<br>Doctor: 12%<br>Nurse or other navigator: 12%	Harrington (2022)
Contact with GP about fatigue, per 1000 patient years	Breast: 57<br>Prostate: 82<br>Colorectal: 63	Heins (2013)
Have you discussed fatigue with your healthcare professional?	Yes: (54.9%)<br>Always: (4.5%)<br>Often: (6%)<br>Sometimes: (10.2%)<br>Never: (24.4%)	La Verde (2002)
Patients talked with HCP about fatigue	Yes: 69%	Chouaïd (2010) & Maloisel (2010)
- I discussed my fatigue with a treating physician at the hospital<br>- I addressed fatigue with my GP	- Yes: 48%<br>- Yes: 36%	Milzer (2022)
Spoken with their doctor about fatigue	Yes: 34%	Passik (2002)
Did discuss fatigue	Yes: 44%	Collins (2008)
Did you talk about fatigue problems with any doctor or nurse (no matter who brought it up)?	Yes: 51%	Van Ryn (2014)

Healthcare use

Health care use for fatigue, defined as visiting a physician or general practitioner, within the last 12 months	Yes: 27.3%	De Ligt (2019)

Number of fatigue statements

Fatigue statements made by	- Physician: 1.2 (sd0.8)<br>- Patient/physician: 2.9 (1.6sd)	Nicklasson (2013)

Last consult

Of the 37 patients, 29 (78%) has discussed fatigue during their last encounter		Tang (2018)
1. Intended to discuss fatigue<br>2. Discussed fatigue	1. Yes: 33%<br>2.<br>- 27% with medical doctor<br>- 5% with nurse coordinator<br>- 16% with nurse practitioner<br>- Total: 48%	Penalba (2019)
Discussed fatigue	Yes: 41%	Donovan (2005)
Fatigue discussed	Yes: 59%	Hilarius (2008)
Fatigue discussed	Yes: 69.5%	Berry (2021)

Recently

One year following cancer diagnosis, contact with GPs was seldom with regard to issues associated with colorectal cancer. Those who were seeing their GP regarding other issues occasionally mentioned issues related to colorectal cancer as an aside. A minority did consult their GP on the ongoing impact of their colorectal cancer, particularly the physical aspects such as fatigue and diarrhoea.		Browne (2011)
Have you discussed with your cancer team about fatigue over the past month?	Yes: 55.6%	Butt (2008)
- Did you discuss fatigue less than 1 month ago?<br>- Did you discuss fatigue more than 1 month ago?	- Yes: 30%<br>- Yes: 15%	Collins (2008)
1. Talk with clinician about fatigue within the past 6 months<br>2. For those without fatigue<br>3. For those with fatigue	1. Yes: 78%<br>2. Yes: 43.5%<br>3. Yes: 91.5%	Smith (2019)

Before treatment

Patients who discussed symptoms of fatigue before chemotherapy	Yes: 91%	Hershman (2008)

Patient experience

Helpful

For those that had fatigue discussions over the past month, were these helpful?	Yes: 82.5%	Butt (2008)

Opportunity

"The majority of the participants found impossible to discuss CRF with a health care provider because of the short duration of medical consultations and/or they do did not know how to approach the topic"		Charles (2021)
2 participants (of 8; 25%) reported difficulty in discussing their symptoms with HCPs, because they felt the busy atmosphere of the centre did not allow HCPs to show the desired interest and allow conversations of this nature to occur		Iguchi (2022)
Patients felt that they did not experience barriers in discussing fatigue with their clinicians		Krok-Schoen (2019)
Did you have enough of an opportunity to explain your concerns about your fatigue to your cancer care team?	Yes: 80.6%	Butt (2008)
Thinks that fatigue is not difficult to raise fatigue with doctors	Yes: 68%	Chouaïd (2010) & Maloisel (2010)

Healthcare professional response

Many participants reported distress when health professionals handled their fatigue with disdain or disinterest.		Bradford (2023)
- "Participants thought that doctors were dismissive when they mentioned fatigue<br>- "Others agreed that healthcare providers were slow to engage in a conversation about fatigue"<br>- Positive experiences with healthcare professionals were also mentioned.		Corbett (2016)
For those few who did raise the topic, the main message they received from the health care professionals was that fatigue was normal and to be expected with cancer treatment.		Fitch (2008)
Patient statements about fatigue were hardly noticed by doctors.		Glaus (2002)
Physicians reactions ranged from stating that there was nothing to be done, to referring patients to another physician.		Iguchi (2022)
Patients did express frustration in the clinicians' responses to their complaints about fatigue. This led patients to reduce efforts to communicate their fatigue. One patient noted, "I'm tired, but unfortunately that's not important to them." Another said, "…I would tell him my symptom but eventually they're going to say, 'It's because of the medicine,' nothing else… why tell them in the first place?"		Krok-Schoen (2019)
1. concerns regarding … fatigue … were dismissed or minimized by clinicians<br>2. The significant mental strain experienced by patients was unacknowledged, dismissed, or minimized by clinicians. Example included fatigue issues for students and or those in high-pressure professions.		O'Neill (2023)
Several CS had experienced a lack of HP understanding		Pearson (2017)
...a perception that HPs did not recognise or prioritise their fatigue		Pearson (2023)
- Doctors thus trivialised patients' CRF experiences by putting them in a cancer context, making it hard for patients to discuss fatigue and making them feel ungrateful or demanding for raising it.<br>- Participants' narratives of CRF were often not recognised or closed off during exchanges with doctors. While participants attributed their fatigue to cancer treatment, doctors frequently distanced the condition from cancer<br>- Eilis' experience of being passed between doctors illustrates how fatigue after cancer falls between the cracks of medical discourse; it is neither in the realm of general medicine nor in the realm of cancer<br>- Many indicated dissatisfaction and frustration at the lack of interest, support and knowledge regarding CRF from the medical community<br>- Doctors' failure to take responsibility for symptoms that participants believe were caused by their treatments is difficult<br>- In their responses to patients' reports of fatigue, some doctors implied that they were naive in thinking that they could be cured of something so serious at no cost. Such responses conveyed the message that one is not entitled to complain about the side effects of life-saving treatments; that fatigue is a small price to pay for surviving<br>-		Pertl (2014)
Another factor that minimized the salience of the fatigue and its distressing effect was that many providers described it as a side effect patients have to tolerate and integrate into their lives.		Siegel (2012)
• In the other cases, participants described a frequent lack of attention or banalization from health care providers regarding CRF."<br>• Several participants expressed the need for listening and recognition of CRF.		Charles (2021)
My fatigue is taken seriously by my treating physician	Yes: 27%	Milzer (2022)
Thinks healthcare professionals are interested in fatigue	Yes: 62%	Chouaïd (2010) & Maloisel (2010)

Figure 2b. Idea webbing and joint display for the patients' perspective on the outcome of communication.

Note: Quantitative data is represented by green shading, while qualitative data is represented by blue shading.

CRF, cancer-related fatigue; CS, consumer; GP, general practitioner; HCP, healthcare professional; HP, health professional.

Healthcare professional - Information

Fatigue information/education

Support groups

Are there opportunities for patients and families to learn about fatigue management at community forums or support group meetings?	Yes: 6%	Miller (2002)

Availability information

1. Is information about fatigue management integrated in existing classes or educational material for patients?<br>2. Does each unit maintain a supply of in institutional-specific brochures of fatigue management?	1. Yes: 0%<br>2. Yes: range 2-11%	Miller (2002)

Understanding fatigue

In Centre 3 the day unit nurses supported, reassured and advised the patient about fatigue and lethargy and provided a clinical explanation for it.	Horne (2014)
Another clinician mentioned he told his patients that the fatigue they feel is not the same fatigue that you feel when you go and work out.	Krok-Schoen (2019)
The men they met were rarely informed about CRF and had little understanding of the condition and its consequences for daily life.	Robertsen (2021)

Patients informed about fatigue

1. Nurses reported that fatigued individuals were provided little education about fatigue and its management.<br>2. Some nurses stated that doctors have adequate information to patients about fatigue. Others described cases where patients lacked sufficient information about fatigue and turned to nurses for support.		Piredda (2007)
The men they met were rarely informed about CRF and had little understanding of the condition and its consequences for daily life.		Robertsen (2021)
1. Patients and families are informed about the importance of managing fatigue?<br>2. Are all patients informed verbally and in an electronic or printed format that effective fatigue management is an important part of their treatment, that it is essential that they report excessive fatigue?	1. Yes: 23%<br>2. 0%	Miller (2002)

Before treatment

Patients are educated before new treatment starts by day chemotherapy nurses. The printed educational information does not include fatigue and were noted as excessive for patients to engage with at times.	Pearson (2023)

Figure 3a. Idea webbing and joint display for the healthcare professionals' perspective on the outcome of information.

Note: Quantitative data is represented by green shading, while qualitative data is represented by blue shading.

CRF, cancer-related fatigue.

Patients - Information

Information & Education

Fatigue as side-effect

Patients were not warned about CRF and did not understand it		Pertl (2014)
Patients were provided with little education on CRF		Piredda (2007)
Patients describe that they were indeed educated about the impending fatigue linked with this treatment. While they may be unable to recall specifics of their teaching, they overwhelmingly claim to know what to expect in regards to fatigue.		Porock (2004)
Patients were informed about HCPs to anticipate fatigue before treatment start		Siegel (2014)
Most patients were informed to expect fatigue as side-effect		Spichiger (2012)
Many patients said fatigue was never mentioned		Corbett (2016)
Fatigue mentioned by doctor when initiating chemotherapy	Yes: 87%	Chouaïd (2010) & Maloisel (2010)
Fatigue discussed as possible side effect	Yes: more than half	Dillon (2003)

Persistence of fatigue

Participants did not feel forewarned about persistence of fatigue after treatment	Corbett (2016)
Patients did not understand their persistent fatigue	Pertl (2014)

Patient impact

When healthcare providers told patients to anticipate fatigue, this seemed to normalize it and decrease distress	Siegel (2012)
Patients that did not feel satisfied with fatigue information, which gave rise to a sensation of abandonment. Those that were satisfied considered the information exchange a positive element	Tolotti (2021)

Initiation of information providing

They did not perceive that nurses had a role in educating them. Patients reported that doctors gave them information about fatigue as a result of their questioning.	Piredda

Information sources

Majority of patients (75%) reported fatigue education solely from provided printed materials	Dolgoy (2020)
Several women used internet, brochures, pamphlets, handouts provided by health practitioners about fatigue	Morgan (2014)
Some reported seeing video, others talked with nurse/physician	Porock (2004)

Satisfaction

Participants indicated it would have been more helpful to receive information about how to handle the fatigue. They thought this was a role health care professionals ought to perform, particularly cancer nurses.		Fitch (2008)
Some patients satisfied with received information, others found information not complete and not enough time dedicated to it		Tolotti (2021)
Cancer team has provided the education/support needed	Yes: 68.5%	Butt (2008)
Feel well informed about fatigue	Yes: 38%	Milzer (2022)
Feel well informed about fatigue	Yes: 27%	Schmidt (2021)
Feel well informed about fatigue	Yes: 25%	Schmidt (2022)

Quality

Patients found existing patient information often too long or detailed for patients with fatigue	Pearson (2023)
Some patients did not find the information provided complete and not enough time dedicated to it	Tolotti (2021)

Figure 3b. Idea webbing and joint display for the patients' perspective on the outcome of information.

Note: Quantitative data is represented by green shading, while qualitative data is represented by blue shading.

CRF, cancer-related fatigue; HCP, healthcare professional.

Healthcare professionals - Screening

Screening

General

Assessing the presence of fatigue was described by nurses as a 'subjective perception', and this generated uncertainty.		Tolotti (2021)
Look for signs of fatigue	3 out of 4	Hingst (2021)
Fatigue screening at regular intervals	Yes: 11%	Luctkar-Flude (2015)

Routinely

The aspects taken into account were how patients carried out their activities of daily living, how they greeted you, and their willingness to talk. The possibility of considering measurable 'objective' data (e.g. blood tests, vital signs, etc.) was also considered important. The collection of these data would lead nurses to include the 'fatigue focus' in the nursing records, where a daily assessment would be made to monitor the course of this symptom.		Tolotti (2021)
Routinely screened or assessed by themselves or others in the workplace	Yes: almost 45%	AL Maqbali (2020)
Fatigue screening at regular intervals	Yes: 11%	Luctkar-Flude (2015)

Tools

The aspects taken into account were how patients carried out their activities of daily living, how they greeted you, and their willingness to talk. The possibility of considering measurable 'objective' data (e.g. blood tests, vital signs, etc.) was also considered important.		Tolotti (2021)
1. Informal interview<br>2. Informal CRF instrument	1. 91.1%<br>2. 9%	AL Maqbali (2020)
1. Personal observation<br>2. Asking about fatigue<br>3. Anamnesis<br>4. Screening instrument and questionnaire	Of those looking for signs of fatigue<br>1. Yes: 37.5%<br>2. Yes: 30.7%<br>3. Yes: 21%<br>4. Yes: 8%, range 0-20%.	Hingst (2021)
Ask patients direct questions about CRF	Nurses: almost 1/3<br>Doctors: (22%)	Pearson (2015)

Figure 4a. Idea webbing and joint display for the healthcare professionals' perspective on the outcome of screening.

Note: Quantitative data is represented by green shading, while qualitative data is represented by blue shading.

CRF, cancer-related fatigue.

Patients - Screening

Patient wishes

Consumers wanted fatigue management as a routine part of cancer care, with screening, assessment and education during regular appointments or treatments such as chemotherapy	Pearson (2017)
"Screening and acknowledgement of fatigue was welcomed by consumers, but it was unusual: 'Fatigue isn't usually mentioned, it's like it's a given and that's that.' (female age 59); 'this is not imaginary, this is debilitating, and it needs attention.' (male age 65)"	Pearson (2023)

Fatigue screening

No time frame/General screening

- "Screening and acknowledgement of fatigue was welcomed by consumers, but it was unusual: 'Fatigue isn't usually mentioned, it's like it's a given and that's that.' (female age 59); 'this is not imaginary, this is debilitating, and it needs attention.' (male age 65)"<br>- Another consumer lowered her fatigue severity rating due to perceptions of staff time pressure		Pearson (2023)
- Many of this study's respondents did not recall having been asked about it.<br>- Occasionally, when oncologists specifically asked about their wellbeing or directly addressed fatigue, patients reported the symptom.<br>- Only three patients complained spontaneously about severe fatigue during their consultations		Spichiger (2012)
My doctor does not ask me about my fatigue, agree or neutral	Yes: 43.3%<br>No: 56.7%	Passik (2002)
Exhaustion or fatigue addressed by treating physician/general practitioner/nurse/psychosocial services	Yes: 62.9%	Schmidt (2021)
Did any doctor or nurse ask you about whether you were having fatigue problems?	Yes: 53%	Van Ryn (2014)
IF a known cancer patient is newly diagnosed with advanced cancer and had 2 or more outpatient visits within 1month of the date of metastases…THEN there should be an assessment of the presence or absence of fatigue within 1mo	Yes: 38%	Walling (2013)

Screening last visit

At your last visit, did a member of your cancer care team ask you about fatigue?	Yes: 52%	Butt (2008)

Screening during treatment

Asked during treatment whether they were fatigued	Yes: 67%	James (2015)
IF a cancer patient is seen for any visit while undergoing chemotherapy at a cancer-related outpatient site…THEN there should be an assessment of the presence or absence of fatigue	Yes: 31.3%	Walling (2013)

Screening consistency

Routinely screened (often or occasionally)	Yes: 48.9%	Pearson (2017)
Periodic check of level fatigue	Yes: 46.7%	Tolotti (2021)
Patient is screened for fatigue using a validated tool at regular intervals throughout the duration of care	Yes: 0%	Wang (2018)
Regularly screened at about half of the appointments or more	Yes: 75.8%	Zucca (2016)
Provider asks patients about fatigue at half of the appointments or more	Yes: 74%	Zucca (2017)

Screening tools

Some participants were asked to rate fatigue on a 100-point scale (range 60-70)		Tang (2018)
- Patient is screened for fatigue using a validated tool upon admission<br>- Patient is screened for fatigue using a validated tool at regular intervals throughout the duration of care	Both 0%	Wang (2018)

Figure 4b. Idea webbing and joint display for the patients' perspective on the outcome of screening.

Note: Quantitative data is represented by green shading, while qualitative data is represented by blue shading.

Healthcare professionals - Assessment

Fatigue assessment

Assessment	Yes: 28.6%	Nurhesti (2020)

Assessment tools

Functional assessment or exercise testing	Yes: 18.5%	Pearson (2015)
1. Doctors and palliative care nurses investigated related symptoms based on personal algorithms or PCOC assessment<br>2. Ward, radiotherapy, chemotherapy and specialist nurses used standard patient health observations and assessments within their scope of practice.		Pearson (2023)
Some of the nurses reported that they did not use the ‘fatigue focus’ but rather a focus on what they define as ‘something similar to it’ (e.g. difficulty moving, or lack of appetite). At times, patients declared that they were feeling weak, describing it as ‘a feeling of uneasiness’.		Tolotti (2021)

Causes

Doctors investigated possible causes including low blood count and depression when the patient complained of fatigue.	Horne (2014)

Objectivity

The possibility of considering measurable ‘objective’ data (e.g. blood tests, vitals signs, etc.) was also considered important.	Tolotti (2021)

Figure 5a. Idea webbing and joint display for the healthcare professionals' perspective on the outcome of assessment.

Note: Quantitative data is represented by green shading, while qualitative data is represented by blue shading.

PCOC, Palliative Care Outcomes Collaboration.

Patients - Assessment

Patient wishes

Consumers wanted fatigue management as a routine part of cancer care, with screening, assessment and education during regular appointments or treatments such as chemotherapy	Pearson (2017)

Fatigue assessment

Patient fatigued/positive screening

How were you diagnosed with CRF?

1. Laboratory tests (blood work)<br>2. Physical examination by HCP<br>3. Questionnaire<br>4. Interview with HCP	1. 16%<br>2. 16%<br>3. 0%<br>4. 64%	Harrington (2022)

Follow-up methods

Participants described having numerous medical tests that aimed to identify a physiological cause for the fatigue. These tests were often invasive and inconvenient.		Corbett (2016)
Global health assessment was expected by some patients, for example, one participant was "waiting for in-depth medical exams to better understand and treat her fatigue		Charles (2021)
1. Focused fatigue assessment<br>2. Risk factors<br>3. Physical examination<br>4. Symptom review<br>5. Laboratory tests<br>Often or occasionally	1. 35.1%<br>2. 23.4%<br>3. 10.7%<br>4. 31.9%<br>5. 20.4%	Pearson (2017)
Interviews and follow-ups to understand the cause of your fatigue	Yes: 33.3%<br>Partly: 13.3%<br>No: 8 53.3%	Tolotti (2021)
Patients who are screened positively for fatigue a focused and comprehensiveness assessment of fatigue is undertaken.	Yes: 0%	Wang (2018)

Outcomes of follow-up

• "Participants described having numerous medical tests that aimed to identify a physiological cause for the fatigue. These tests were often invasive and inconvenient. Importantly, they were also seen to reduce the validity of fatigue as an issue in and of itself, which led to feelings of confusion, uncertainty, and fear."<br>• Participants voiced frustration that a label or definition of CRF was not provided to them. Acknowledgement of fatigue was viewed as a tool for acceptance that could reduce worry about the symptom and would justify the "right to be tired"	Corbett (2016)
Participants' indicated that doctors dismissed fatigue when there was no clear diagnosis	Pertl (2014)
Another said that a physical examination helped to reassure her about the objective existence of CRF: "When I go to my doctor, I'm happy when she measures my blood pressure and she tells me "you have ten," because sometimes you wonder if it's not in your head."	Charles (2021)

Figure 5b. Idea webbing and joint display for the patients' perspective on the outcome of assessment.

Note: Quantitative data is represented by green shading, while qualitative data is represented by blue shading.

CRF, cancer-related fatigue; HCP, healthcare professional.

Healthcare professionals - Advice

Considered effective advice

- Advocated increased rest or sleep as being beneficial in managing fatigue.<br>- A common belief among nurses was that there was little to do but rest.	Piredda (2007)

Advice

Type of advice

Day unit nurses discussed the role of activity in fatigue management at all time points in the transplant journey.		Horne (2014)
Clinicians acknowledged that fatigue is a common symptom in their practice and instructed patients to engage in regular activities as much as possible to avoid worsening the symptom.		Krok-Schoen (2019)
'Some staff e.g. nurses, provided individual energy management education as part of usual care.		Pearson (2023)
1. Nurses described interventions such as encouraging patients to take care of their personal hygiene and to get out of bed.<br>2. However, nurses reported that these types of interventions were implemented on their own initiative. Therefore, there were various ways of addressing fatigue, some nurses focused more on encouraging patient mobility and others on nutrition, almost as if the interventions depended on the nurses' individual sensations or a personal way of viewing the problem.		Tolotti (2021)
Implemented the fatigue activity part of breast cancer management guidelines: Recommend engaging in 30 min of moderate-intensity physical activity most days unless contraindicated (eg, fast walking, cycling, swimming, and resistance training)	Yes: 37.8%	Luctkar-Flude (2015)

Figure 6a. Idea webbing and joint display for the healthcare professionals' perspective on the outcome of advice.

Note: Quantitative data is represented by green shading, while qualitative data is represented by blue shading.

Patients - Advice

Fatigue advice/recommendations

Patient needs

Several CS noted that CRF is an individual experience and that management needs to be tailored to fatigue related limitations.	Pearson (2017)
Patients wanted to receive advice from nurses on how they could manage fatigue but this did not always happen	Tolotti (2021)

Actions initiated without advice

Several participants tried to balance periods of activity and rest to manage their fatigue, although they tried this strategy without being advised by anyone to do so	John (2010)

Received advice/recommendations

Fatigue level related

Patients not fatigued

Explained what to do if the symptom started, became worse, or returned within the past 6 months?	Yes: 160 (31.5%)<br>No: 348 (68.5%)	Smith (2019)

Changing fatigue levels

Explained what to do if the symptom started, became worse, or returned within the past 6 months?	Yes: (61%)	Smith (2019)
Did any doctor or nurse give you instructions about what to do if the fatigue problems started up, got worse, or came back?	Yes: (28%)	Van Ryn (2014)

Fatigued patients

1. Given advice to help cope with symptoms<br>2. Been advised about fatigue before starting treatment<br>3. Been advised about fatigue during treatment	1. Yes: 55%<br>2. Yes: 86%<br>3. Yes: 72%	James (2015)
Explained what to do if the symptom started, became worse, or returned within the past 6 months?	Yes: 1053 (72.8%)<br>No: 394 (27.2%)	Smith (2019)

General

1. Patients received no information or guidance from their physicians on how to manage fatigue.<br>2. Patients experienced that support or providing information on managing fatigue is not standard practice.<br>3. Professionals did not offer any help for managing fatigue		Glaus (2002)
Little support was available, advice was lacking. Example included fatigue missed for students or those in high-pressure professions.		Oneill (2023)
1. Patients recognized that they had not received any help or advice on how to manage this symptom<br>2. Although doctors seemed to inform some patients about the likely occurrence of fatigue, neither doctors nor nurses gave advice about how to manage it.		Piredda (2007)
1. Advice from health professionals on how to deal with fatigue was markedly absent from patients' accounts<br>2. No patients reported any counselling by health professionals on fatigue management strategies.		Spichiger (2012)
1. Patients wanted to receive advice from nurses on how they could manage fatigue but this did not always happen, and according to the patients this was because nurses did not have enough time or had to give the priority to patients who were 'feeling worse than them'.<br>2. Some patients reported that they had been encouraged to walk, and considered physicians and nurses as their "sports coach". Other patients reported that although they did speak to nurses, they did not receive any advice on how to manage fatigue. They remember that sometimes nurses remained in silence, an attitude that was interpreted as 'they are reflecting, they are thinking about it'.		Tolotti (2021)
Fatigue advice given	Yes: 31.9%<br>No: 67.1%	Blaney (2011)
1. Treatment/advice/help/action against fatigue (but not specified help from who, also includes family)<br>2. Advice from a healthcare professional	1. Yes: 46%<br>No: 54%<br>2. Yes: 15%	Collins (2008)
Discuss fatigue relieving strategies	29%	Dillon (2003)
Received recommendations	Yes: 44%	Donovan (2005)
Received support or advice	Yes: 32%	Jenkins (2013)
Patient is informed of the strategies to manage CRF	Yes: 0%	Wang (2018)

Helpful

They reported that they had been advised by their healthcare providers to rest or sleep to manage fatigue, but this was not effective	John (2010)
Several CS commented that they had not received helpful advice from their HPs regarding fatigue management, suggesting a lack of up-to-date knowledge about CRF	Pearson (2017)

#recommendations

Mean recommendations of medical staff for fatigue	11.63 (7.55 SD) range 0-25	Passik (2002)

Type of advice

Of those that spontaneously mentioned they have raised the problem with a healthcare provider "only two participants had been informed about the benefit of exercise, and in one case, the participant was already physically active.		Charles (2021)
1. They reported that they had been advised by their healthcare providers to rest or sleep to manage fatigue, but this was not effective<br>2. The comment of one participant seemed to represent the common experience of all participants, "When I told the doctor about being so tired or wiped out, he said, 'Go to bed.'"		John (2010)
Some patients reported that they had been encouraged to walk, and considered physicians and nurses as their "sports coach".		Tolotti (2021)
1. Exercise/keep active<br>2. Rest/sleep<br>3. Pacing<br>4. Other	1. 68.0%<br>2. 64.5%<br>3. 57.4%<br>4. 163%	Blaney (2011)
Were you given suggestions, other than taking medicines, by your cancer care team on how to handle your fatigue?	Yes: 41.9%	Butt (2008)
Recommendations received:<br>1. Rest and relaxation<br>2. Nothing/no recommendation/prescription<br>3. Diet/nutrition<br>4. Exercise<br>5. Transfusion<br>6. Reduce activity<br>7. Vitamins<br>8. Prescription drug-iron<br>9. Antidepressants<br>10. Other prescription drugs<br>11. Other prescription drugs-EPO<br>12. Other<br>13. Don't know/not stated	Recommendations received:<br>1. 32%<br>2. 27%<br>3. 9%<br>4. 7%<br>5. 5%<br>6. 5%<br>7. 4%<br>8. 3%<br>9. 3%<br>10. 2%<br>11. n=1;0%<br>12. 5%<br>13. 7%	Dillon (2003)
1. Information/suggestions on how to subdivide the day<br>2. Information/suggestions on how to reduce efforts<br>3. Information/suggestions on how to ask others to do some activities<br>4. Information/suggestions on how to measure fatigue by yourself<br>5. Reassurance and/or psychological support<br>6. Encouraged to distract yourself<br>7. Help to keep activity levels to the minimum	1. Yes/partly: 54.5%<br>No: 45.5%<br>2. Yes/partly: 53.8%<br>No: 46.2%<br>3. Yes/partly: 61.5%<br>No: 38.5%<br>4. Yes/partly: 45.5%<br>No: 54.5%<br>5. Yes/partly: 71.4%<br>No: 28.6%<br>6. Yes/partly: 53.3%<br>No: 46.7%<br>7. Yes/partly: 73.3%<br>No: 26.7%	Tolotti (2021)
Patient is informed of the benefits and encouraged to undertake physical activity	Yes: 3%	Wang (2018)

Figure 6b. Idea webbing and joint display for the patients' perspective on the outcome of advice.

Note: Quantitative data is represented by green shading, while qualitative data is represented by blue shading.

CRF, cancer-related fatigue; CS, consumer; EPO, erythropoietin; HCP, healthcare professional; HP, health professional.

Healthcare professionals - Treatment

Treatment

Beliefs

Fatigue was described as a multifactor symptom, which nurses considered complicated to manage	Tolotti (2021)
Nurses: 70% beliefs fatigue can be relieved.<br>Physicians: less than 50%	Dillon (2003)

Considered effective treatments

1. Transfusions and specific drugs<br>2. Only transfusions<br>3. Only drugs<br>4. Treating the underlying disease	1. Yes: 47.7%<br>2. Yes: 33.3%<br>3. Yes: 10.3%<br>4. Yes: 6.3%	La Verde (2002)
Doctors/nurses<br>1. Activity management<br>2. Exercise<br>3. Mind-body<br>4. Nutrition<br>5. Pharmacological<br>6. Psycho-education<br>7. Rehabilitation<br>8. Support/expressive<br>9. Symptom management	Doctors/nurses<br>1. 10%/27%<br>2. 40%/34%<br>3. 10%/9%<br>4. 3%/23%<br>5. 70%/5%<br>6. 30%/18%<br>7. 40%/11%<br>8. 0%/9%<br>9. 20%/32%	Pearson (2015)

Treatments implemented/availability

Does your practice routinely implement interventions for cancer-related fatigue (e.g., exercise program, nutritional consultation, rehabilitation, psychosocial interventions, psychostimulants, according to the NCCN after ruling out non-cancer comorbidities (e.g., endocrine, cardiac or pulmonary dysfunction) that may contribute to symptoms of fatigue?	Yes: 66%	Ruiz (2014)
Available supportive care and activities for the management of fatigue	Yes: 80%	Simon (2020)
Implemented the fatigue activity part of breast cancer management guidelines: Recommend engaging in 30 min of moderate-intensity physical activity most days unless contraindicated (eg, fast walking, cycling, swimming, and resistance training)	Yes: 37.8%	Luctkar-Flude (2015)

Treatment following reporting/screening

Patients who experience fatigue and report it to their nurse or physician who then receive treatment	Half of the patients	Dillon (2003)
Percentage patients to be treated for fatigue according to the healthcare professionals	Between 0-20%: (7.7%)<br>Between 21-40%: (4.0%)<br>Between 41-60%: (13.3%)<br>Between 61-80%: (20.7%)<br>More than 80%: (36.0%)	La Verde (2002)

No availability

The clinical psychology department provided CBT programs for insomnia, anxiety and fear of cancer recurrence but none specifically targeted to cancer fatigue, and none existed in the community.	Pearson (2023)

Recommended treatments

1. Physical activity<br>2. Psychosocial interventions<br>3. Mind-body interventions	1. Yes: 60%<br>2. Yes: 35%<br>3. Yes: 25%	Martin (2020)
Referrals to OT and optimisation clinic were made by some nurse specialists.		Pearson (2023)
1. Exercise<br>2. Rest<br>3. Prescription drugs<br>4. Diet<br>5. Complementary therapy<br>6. EPO<br>7. Other	1. Physicians: ~50%<br>Nurses: ~65%<br>2. Physicians: ~80%<br>Nurses: ~95%<br>3. Physicians: ~35%<br>Nurses: ~10%<br>4. Physicians: ~35%<br>Nurses: ~55%<br>5. Physicians: ~30%<br>Nurses: ~60%<br>6. Physicians: ~5%<br>Nurses: ~2%<br>7. Physicians: ~15%<br>Nurses: ~15%	Dillon2003
If you notice fatigue, what do you do?<br>1. Document presence and severity<br>2. Recommend rehabilitation sport<br>3. Recommend physical activity<br>4. Refer to colleages in Psychology<br>5. Refer to PIZ<br>6. Recommend pharmaceuticals<br>7. Other	If you notice fatigue, what do you do?<br>1.Yes: 17.0%<br>2. Yes: 4.5%<br>3. Yes: 20.2%<br>4. Yes: 29.6%<br>5. Yes: 19.4%<br>6. Yes: 5.3%<br>7. Yes: 4.0%	Hingst (2021)

Considering other specialities

Oncology nurses frequently called for additional assistance of a psychological nature for the men to better support them and their families	Robertsen (2021)
Other than the interventions made by the nurses, there was also an 'organizational' aspect regarding the procedure to request the specific intervention of other health professionals, such as physiotherapists, dieticians or psychologists.<br>2. However, nurses reported that these types of interventions were implemented on their own initiative. Therefore, there were various ways of addressing fatigue, some nurses focused more on encouraging patient mobility and others on nutrition, almost as if the interventions depended on the nurses' individual sensations or a personal way of viewing the problem.	Tolotti (2021)

Figure 7a. Idea webbing and joint display for the healthcare professionals' perspective on the outcome of treatment.

Note: Quantitative data is represented by green shading, while qualitative data is represented by blue shading.

CBT, cognitive behavioural therapy; EPO, erythropoietin; NCCN, National Comprehensive Cancer Network; OT, occupational therpay; PIZ, patient information center.

Patients - Treatment

Satisfaction

I receive a good therapy for my fatigue	Yes: 13%	Milzer (2022)
1. Did any doctor or nurse give you the help you wanted for fatigue?<br>2. During the Last 6 Months, Did a Physician or Nurse Give You the Help You Wanted to Take Care of Your fatigue?	1. Yes: (20%)<br>2. Yes: 280 (41%)<br>No: 408 (59%)	van Ryn (2014)

Awareness

..a large majority of the participants did not have a picture of what educational or psychosocial interventions targeting CRF could be like.	Charles (2021)
They did not receive a strong indication that interventions existed to help them cope with the side effects	Fitch (2008)
1. A couple of women were aware of medication that they could receive that may assist with their fatigue.<br>2. Some African American women reported that they did not receive information about medications that would assist them with the side effects of fatigue.	Morgan (2014)

Fatigue treatment

Ask for treatment

Asked for treatment but nothing was recommended by oncologist	Yes: 16.8%	Diaz (2008)

Treatment offered

1. Treatment for fatigue offered by physician<br>2. Received treatment for fatigue	1. (30%)<br>2. (22%)	Simon (2020)
My doctor has not offered me any intervention for fatigue.	Agree: 46.7%<br>Neutral: 19.9%<br>Disagree: 34.1%	Passik (2002)
Offered treatment by doctor	Yes: 50%	La Verde (2002)

Discussion

1. Several participants engaged in moderate exercise such as walking three times a week prior to their breast cancer diagnosi s. These women asked their physicians if they could continue exercising while going to treatment and their physicians responded positively.<br>2. Several African American women reported utilizing alternative methods such as vitamin supplements, herbal remedies, and natural teas while undergoing breast cancer treatment. These participants reported obtaining permission from their physicians to use alternative therapies in conjunction with their routine breast cancer treatment.<br>3. African American women discussed using conventional medicines prescribed by their physicians to assist them with fatigue related to cancer treatment. These women tended to describe that the medications prescribed to combat fatigue were not very helpful.		Morgan (2014)
1. Did discuss treatments for the NCCN treatment category (regardless of provided or not)<br>2. Discussed treatment, but no specific counselling, instruction, or prescription provided	1. General strategies: (42.80%)<br>Activity enhancement: (26.25%)<br>Psychosocial strategies: (26.25%)<br>Pharmaceuticals: (48.75%)<br>2. General strategies: 42 (26.50%)<br>Activity enhancement: 24 (15.00%)<br>Psychosocial strategies: 26 (16.25%)<br>Pharmaceuticals: 19 (11.88%)	Cheville (2013)
Discussed fatigue treatments with their physicians	Yes: 72.9%	Diaz (2008)
Were you told there is a treatment option for CRF?	Yes: 20%	Harrington (2022)
Patients who discussed treatments for adverse event before chemotherapy	Yes: 76%	Hershman (2008)

Treatment received

Professionals did not offer any help for managing fatigue, and in contrast to English patients, no referral to experts was made in Swiss.		Glaus (2002)
Received treatment for fatigue	Yes: fewer than 17% (excluding pharmaceuticals)	Cheville (2013)
Treatment/advice/help/action against fatigue (but does not specify from whom, also includes family)	Yes: 46%<br>No: 54%	Collins (2008)
Received treatment for fatigue	Yes: 61.2%	Díaz (2008)
Did you receive treatment for CRF?	Yes: 16.0%<br>No: 60.0%<br>Unsure: 20.0%	Harrington (2022)
Medical personnel provided intervention to manage fatigue	Yes: 54.8%	Rau (2020)
Received treatment for fatigue	Yes: 22%	Simon (2020)

Recently received

Have you received any treatment for fatigue over the past month?	Yes: 12.1%	Butt (2008)

Working treatment

If you have started medicines or other treatments for your fatigue, has anyone from your cancer care team asked you how well the treatment was working? Yes:	Yes: 6.6%	Butt (2008)

Type of treatment

Have you been sent to see another health care provider for treatment for your fatigue?	Yes: 4.3%	Butt (2008)
Reported types of care included medical treatment	Yes: 15%	Collins (2008)
1. General strategies<br>2. Activity enhancement<br>3. Psychosocial strategies<br>4. Pharmaceuticals	1. 16.30%<br>2. 11.25%<br>3. 10.00%<br>4. 36.88%	Cheville (2013)
Received medication prescription	Yes: 11.1%	Donovan (2005)
Did you receive treatment for CRF?<br>1. Medication<br>2. Education<br>3. Supervised exercise; yoga; acupuncture; other	1. Yes: 75%<br>2. Yes: 50%<br>3. 0%	Harrington (2022)
For treatment, it is unsure which non-pharmacological treatments are self-initiated and which ones were recommended by the medical professionals. For pharmacological treatments, interventions consisted of<br>1. Blood transfusion<br>2. Steroids<br>3. Hormone<br>4. Sleep & sedative drugs<br>5. Methylphenidate	1. 11.5%<br>2. 6.2%<br>3. 1.4%<br>4. 1.3%<br>5. 0.3%	Rau (2020)
1. Exercise/physical activity<br>2. Relaxation<br>3. Treatment of metabolic disorders<br>4. Homeopathic or herbal drugs<br>5. Psychological support<br>6. Psychopharmaceuticals<br>7. Yoga<br>8. Treatment of anaemia<br>9. Cessation of drugs<br>10. Acupuncture or acupressure<br>11. Sleep counselling or therapy	1. 67.7%<br>2. 23.9%<br>3. 23.5%<br>4. 14.5%<br>5. 13.0%<br>6. 12.3%<br>7. 8.6%<br>8. 5.3%<br>9. 4.1%<br>10. 4.0%<br>11. 3.6%	Schmidt (2021)
1. Involve specialists: for mobilization (physiatrists, physiotherapists)<br>2. Involve specialists: with massages<br>3. Involve specialists: for psychological support (psychologists etc)<br>4. Involve specialists: for nutritional support (nutritionists, dietitians, etc)<br>5. Interventions to facilitate sleep/rest	1. Yes: 53.3%<br>2. Yes: 7.1%<br>3. Yes: 14.3%<br>4. Yes: 26.7%<br>5. Yes/partly: 60.0%	Tolotti (2021)

Timely treatment

Not receiving a timely intervention for fatigue	Yes: 45%	Rau (2020)

Figure 7a. Idea webbing and joint display for the healthcare professionals' perspective on the outcome of treatment.

Note: Quantitative data is represented by green shading, while qualitative data is represented by blue shading.

CRF, cancer-related fatigue; NCCN, National Comprehensive Cancer Network.

Patients & Healthcare professionals - Documentation

Documentation

Treatment

1. Pharmacologic intervention<br>Referrals made to<br>2, OT/PT<br>3, Nutritionist<br>4, Psychosocial support<br>5, Support groups	1. Yes: 2.90%<br>2. Yes: 0%<br>3. Yes: 1.45%<br>4. Yes: 2.90%<br>5. Yes: 0%	Borneman (2007)
Recorded advice to manage fatigue	Yes: 3%	James (2015)
Of those with self-reported fatigue (n=35), fatigue management documented	Yes: 20%	Bock (2012)
Fatigue was a contributing or a sole factor for hospital admittance in eight patients . Treatment plan for fatigue prepared	Yes: n=1	Tarp (2023)

Therapeutic measures taken

Diagnostic and therapeutic actions taken during consultation	0.29 (sd0.79)	Nicklasson (2013)

Patient education

1. Regarding fatigue patterns<br>2, Regarding daily self-monitoring<br>3, Regarding energy conservation<br>4, Regarding activity enhancement<br>5, Regarding sleep hygiene<br>6, Regarding restorative therapy<br>7, Regarding stress management<br>8, Regarding relaxation methods	For all: Yes: 0%	Borneman (2007)

Documentation forms

1. Do current nursing documentation forms screen for fatigue and provide for the ongoing recording of assessment, interventions and trends of fatigue relief?<br>2. Do current physician documentation forms screen for and assess fatigue management?<br>3. Does your method for fatigue documentation place fatigue in a highly visible and prominent position that encourages regular review by all disciplines?	1. Yes: 0-6%<br>2. Yes: 8% of medical nurses, 0% for all other specialties<br>3. No scores reported, but indication that this is poorly.	Miller (2002)

Any sort of information/documentation

Fatigue-related information in electronic health records 3/6/12/24 months before last data collection	Yes: 0% (for all)	Carreira (2022)
Fatigue documented in medical records (nurses only)	Yes: range 0-7%	Carpenter (2008)
Any documentation of fatigue in physician records	Yes: 16.7%	Marino (2020)
1. Documentation fatigue in physician records<br>2. Documentation fatigue in nurses records	1. Yes 21.1%<br>2. Yes 7%	Tolotti (2021)

Assessment of fatigue

Symptoms captured by PRO assessments like sadness, loss of appetite, fatigue, trouble sleeping, and dry mouth and functional restrictions were not documented regularly in the medical records		Schmidt (2016)
1. Focused history conducted for moderate to severe fatigue<br>2. Review of systems performed for moderate to severe fatigue<br>3. In-depth assessment conducted for moderate to severe fatigue	1. Yes: 1.45%<br>2, Yes: 18.84%<br>3, 1.45%	Borneman (2007)

Diagnostic measures taken

Diagnostic and therapeutic actions taken during consultation	0.29 (sd0.79)	Nicklasson (2013)

Fatigue as symptom/Fatigue screening

1. Fatigue rating documented<br>2. Screening for fatigue at visit<br>3. Screening for fatigue is consistent	1. Yes: 2.9%<br>2. Yes: 23.19%<br>3. Yes: 8.70%	Borneman (2007)
1. Notes recording patient experiencing fatigue, lethargy, or feeling tired on at least one occasion<br>Of these patients<br>2. Fatigue noted more than once	1. Yes: 43%<br><br>2. Yes: 28%	James (2015)
1. Fatigue as gradable symptom mentioned in medical record<br>2. Fatigue mentioned in medical record, not gradable<br>3. Fatigue not mentioned in medical record<br>Note: the data has been collected for 10 randomly selected patients in the NSA group (for 4 investigators each)	1. Yes: 10.6%<br>2. Yes: 13.3%<br>3. Yes: 76.3%	Leung (2022)
1. Fatigue mentioned in medical records that the symptom is present<br>2. Fatigue mentioned in medical records that the symptom is not present<br>3. Fatigue not mentioned	1. Yes: 43%<br>2. 0%<br>3. 57%	Tarp (2023)
Assessed at least once by the treating oncologist.	Yes: 68.8%	Alcalde-Castro (2020)
Symptom fatigue assessed by doctor	Yes: 60%	Mark (2008)
Assessing the presence of fatigue was described by nurses as a 'subjective perception', and this generated uncertainty. The aspects taken into account were how patients carried out their activities of daily living, how they greeted you, and their willingness to talk. The possibility of considering measurable 'objective' data (e.g. blood tests, vital signs, etc.) was also considered important. The collection of these data would lead nurses to include the 'fatigue focus' in the nursing records, where a daily assessment would be made to monitor the course of this symptom		Tolotti (2021)

Patients without fatigue

1. Patient not fatigued, no documentation by physician<br>2. Patient not fatigued, documentation by physician<br>Note: 6,4% of the whole sample including those with fatigue. 100% of patients without fatigue had no documentation	1. Yes: 6.4%<br>2. 0%	Marino (2020)

Patients with fatigue

1. Self-reported fatigue but fatigue not documented in clinical notes<br>2. Self-reported fatigue and documented in clinical notes	1. Yes: 51%<br>2. Yes: 49%	Bock (2012)
1. Patient fatigued, no documentation by physician<br>2. Patient fatigued, documentation by physician<br>Note: 1&2 are based on all patients, including those without fatigue. Of those with fatigue: 3&4	1. Yes: 76.8%<br>2. Yes: 16.7%<br>3. Yes: 82%<br>4. Yes: 18%	Marino (2020)
Of those with clinically significant fatigue (moderate to severe) (n=300; 70%)<br>1. Fatigue mentioned in clinical notes<br>2. Appraisal of fatigue consisted with self-rating fatigue<br>Of those with severe fatigue (n=167; 39%)<br>3. Fatigue mentioned in clinical notes<br>Many clinical notes only listed fatigue as a possible treatment side effect.<br>4. In those were fatigue was mentioned (n=54): a fatigue grade	1. 54 (17%)<br>2. Yes: 1/3<br>3. 29 (17%)<br>4. 9 (17%)	Pearson (2021)

Figure 8. Idea webbing and joint display for the patients' and the healthcare professionals' perspective on the outcome of documentation.

Note: Quantitative data is represented by green shading, while qualitative data is represented by blue shading.

NSA, non-structured assessment; OT, occupational therapist; PRO, patient-reported outcome; PT, physical therapist.
