# Supplementary material for: The gap between guidelines and clinical practice in cancer-related fatigue management: a mixed-methods systematic review
Source: J Cancer Surviv. 2025 Jul 18;20(Suppl 1):1220–39. doi: 10.1007/s11764-025-01866-6 (PMC13676525; doi:10.1007/s11764-025-01866-6)
Supplement: Supplementary file 3 [file 11764_2025_1866_MOESM3_ESM.pdf]

<u>Supplementary Materials: File 3</u>

The Gap Between Guidelines and Clinical Practice in Cancer-Related Fatigue Management: A Mixed-Methods Systematic Review.

Journal of Cancer Survivorship

Marijn Witziers[a], Anne Looijmans[a], Ning Jia[a], & Mariët Hagedoorn[a]

[a], Department of Health Psychology, University Medical Centre Groningen, University of Groningen, the Netherlands

Correspondence: Marijn Witziers

Email: m.witziers@umcg.nl

Table 1: The main characteristics of the included studies.

Author	Study methods	Included patients		Included healthcare professionals	
		Characteristics	Outcome	Characteristics	Outcome
Quantitative non-intervention studies					
Al Maqbali et al. (2020) (1)	The objective was to assess HCPs' knowledge of and current practices in managing CRF in Oman. The survey was			138 HCPs (73.2% female). Most common age range 25-34 years	Guideline usage, screening, and facilitators.

	based on Pearson et al. (2015), the PFS, and the City of Hope Quality of Life model.			(49.3%). Common professions: nurses (74.6%), GPs (8%), and oncologists (5.8%).	
Alcade-Castro et al. (2020) (2)	The goal was to evaluate symptom management in advanced cancer patients. The chart review focused on frequency of symptom assessments and the reasons for referrals to supportive and palliative care within the first year post-diagnosis.	77 patients (45.5% female, mean age 62 years, range 47-77). Common primary disease sites: gastrointestinal (53.2%) and genitourinary (20.8%). All patients had advanced disease	Documentation.		
Blaney et al. (2013) (3)	The study aimed to explore exercise-related barriers, facilitators, and preferences among cancer survivors. The survey also included sections on CRF management and related advice. CRF was measured using the MFSI-SF.	456 patients (76.0% female, median age 61 years, IQR 15). Common cancers: breast (64.4%) and prostate (12.4%). Most patients were in stages 1 (18.3%), 2 (21.9%), or 3 (16.7%).	Advice.		

Bock et al. (2012) (4)	The study sought to investigate the impact of a web-based health questionnaire. A survey and chart review tracked the number of symptoms reported by patients, documented by HCPs, and addressed during consultations.	106 female patients (mean age 56.9 years, range 32-85). All had breast cancer in stages 1-3.	Documentation		
Borneman et al. (2007) (5)	As part of a five-year prospective trial, this study compared usual care for fatigue with evidence-based guidelines by conducting chart reviews. Fatigue was measured using the FIS and the PFS.	69 patients (62.3% female, mean age 60.6 years, SD 12.52). Cancer types: breast (38%), colon (23%), lung (25%), and prostate (14%). Most had stage 3 (44%) or stage 4 (38%).	Documentation		
Butt et al. (2008) (6)	This longitudinal study aimed to survey cancer patients about their symptoms. Interviews explored patients' interactions with their healthcare team, satisfaction with clinical services, and approaches to	99 patients (71.7% female, mean age 57.2 years, SD 12.4). Common cancers: breast (28.3%), colorectal	Communication, information, screening, advice, and treatment.		

	symptom management. Fatigue was measured using the FACT-F.	(17.2%), and lung (13.1%). 41.1% had metastatic disease.			
Cardoso et al. (2023) (7)	The objective was to examine HCPs' and patients' perspectives, through a survey, on the importance of QoL discussions and the impact of side-effects on QoL during cancer treatment.	467 patients (99% female, mean age 46.6 years, SD 10.7). All had breast cancer, 47% in stage 3b, and 49% in stage 4.	Communication		
Carreira et al. (2022) (8)	Through a survey and a medical chart review, this study aimed to compare PROs with data recorded in primary care electronic health records. Fatigue was assessed using the QLACS tool.	356 female patients. Most were aged 60-69 years (36.5%) or ≥70 years (34.6%). All patients had breast cancer, most had localized (54.8%) or regional metastases (43%) at diagnosis.	Documentation.		
Cheville et al. (2013) (9)	The focus of this study was to evaluate how frequently stage IV cancer patients received guideline-endorsed fatigue	160 patients (49.4% female, mean age 66.8, SD 10.7). Cancer types: breast (25%),	Treatment.		

	treatments. In interviews, patients were asked about the care they received from their oncology team concerning different fatigue treatment domains.	lung (25%), colon (25%), and prostate (25%). All in stage 4.			
Chouaïd et al. (2010) & Maloisel et al. (2010) (10, 11)	The study evaluated and compared patients' and HCPs' perceptions of fatigue and chemotherapy-induced anemia management through surveys.	300 patients (51% female, mean age 60.8 years, SD 11.6). Common cancers: non-myeloid hematological malignancies (27%), colorectal (24%), lung (24%), and breast (23%).	Communication and information.		
Collins et al. (2008) (12)	This study explored the experiences of pain and fatigue in patients with incurable cancer. Based on the questionnaire by Osse et al. (2002), patients were interviewed about symptom discussions with HCPs and current management strategies. Fatigue	125 patients (52% female, mean age 63.3 years, SD 10.5). Common cancers: lung (41%), breast (25%), and colorectal (18%). All had advanced disease.	Communication, advice, and treatment.		

	was assessed using the EORTC QLQ-C30.				
De Ligt et al. (2019) (13)	The goal was to examine treatment-related health problems in breast cancer survivors up to five years post-treatment. A self-administered survey covered reported health issues and associated healthcare utilization.	404 female patients (mean age 62 years, SD 10.9). All had breast cancer, most in stage 1 (46%) or stage 2 (43%).	Communication.		
Diaz et al. (2008) (14)	Through a survey, the functional and psychological impact of fatigue was explored. Patients reported on discussions about and their experience with fatigue treatment. Fatigue was measured using a 10mm VAS.	505 patients (55.2% female, mean age 58.8 years, SD 11.7). Common cancers: breast (26.5%), gastrointestinal (20.8%), and lung (14.5%).	Communication, treatment.		
Dillon & Kelly (2002) (15)	A patient survey assessed the nature and impact of fatigue on patients' lives, while HCPs were surveyed to evaluate their	143 patients (32% female, mean age 58.3 years, range 21-82). Common cancers: gastrointestinal (36%),	Communication, information, and advice.	269 HCPs: nurses (n=160) and physicians (n=109).	Communication and treatment.

	perceptions of fatigue and current management practices in Ireland.	gynecological (27%), and breast (15%).			
Donovan & Ward (2005) (16)	This study aimed to describe women's representations of fatigue and coping efforts related to fatigue. A survey was used to assess fatigue representations using the SRQ. Coping efforts were evaluated through communication with HCPs, adherence to treatment recommendations, and self-directed strategies.	27 female patients (mean age 58.19 years, SD 10.12). All had gynecological cancer.	Communication, advice, and treatment		
Harrington et al. (2022) (17)	This study examined physical therapists' CRF screening practices and cancer survivors' knowledge and experiences with CRF. The patient survey included questions on CRF diagnosis, discussions with HCPs, treatment options, and patients' perceptions of CRF.	61 patients (90.2% female, mean age 57.2 years, SD 12.9). Common cancers: breast (62.3%) and gynecological (13.1%).	Communication, assessment, and treatment.		

Heins et al. (2013) (18)	The aim of this study was to explore the diseases and complaints for which cancer patients contacted their GP 2–5 years after diagnosis. Electronic health records were reviewed to identify fatigue and determine whether it was discussed during consultations.	2,246 patients (67.3% female, mean age 63 years, SD 13.21). Common cancers: breast (55.9%), prostate (22.4%), and colorectal (21.7%).	Communication.		
Herschman (2008) (19)	The study examined patients' perceptions of the physician-patient relationship and communication regarding cancer-related issues, particularly adverse events. A survey, based on questions from the Harris Poll's question bank, focused on symptom discussions, symptom management, and treatment-related concerns.	508 patients (64% female, 50% aged ≥65 years).	Communication and treatment.		
Hingst et al. (2021) (20)	The study assessed awareness of a fatigue seminar, its recommendation to patients, and reasons for missed referrals.			115 HCPs.	Screening and treatment.

	Additionally, the survey covered fatigue knowledge, diagnostic approaches, treatment, and perceived responsibility in fatigue management.				
James (2015) (21)	The first part of this study aimed to determine the incidence and impact of CRF in patients receiving radiotherapy or systemic anti-cancer therapy. Medical charts were reviewed to identify CRF symptoms and whether HCPs provided for managing these symptoms.	68 patients (most aged 51-75 years, 65%). Common cancers: breast (51%), prostate (15%), and colorectal (12%).	Documentation		
	The second part, based on the results of the chart review, examined through a survey whether patients had been informed about potential CRF, monitored for symptoms, and got offered advice or help. The EORTC QLQ-FA13 was used to measure patients' CRF.	146 patients (aged 58-76 years). Common cancers: breast (48%), prostate (19%), and bowel (11%).	Advice and screening		

Jenkins et al. (2013) (22)	The study examined the experiences and preferences of women with advanced ovarian cancer regarding treatment and care, comparing them with oncologists' practices in the UK. Structured patient interviews covered treatment side effects and support received. Fatigue was assessed using the EORTC QLQ-C30.	202 female patients (mean age 63.5 years, SD 9.6). All patients had ovarian cancer, with 60% in stage 3 and 31% in stage 4.	Advice.		
La Verde & Arienti (2002) (23)	A survey was conducted to enhance understanding of fatigue in Italy. The questionnaire for HCPs assessed their views on the prevalence, causes, impact, and management of fatigue in hematologic cancer patients. The patient questionnaire focused on personal experiences with fatigue, its effects on daily life, and patients' interactions with HCPs regarding fatigue treatment.	300 patients (47.3% female, mean age 52 years). All had hematologic malignancies, with non-Hodgkin's lymphoma (36%) and Hodgkin's lymphoma (18.3%) being most common.	Communication and treatment.	300 HCPs. Common age ranges: 36-45 years (40%) and 46-55 years (37%). All were hematologists.	Treatment.

Leung et al. (2022) (24)	This observational study evaluated the feasibility of implementing a toxicity screening tool to manage cancer-related symptoms. Chart reviews provided insights into the documentation and severity of symptoms experienced by patients.	37 male patients (mean age 66.35 years, SD 7.87). All had prostate cancer, with 59.4% in stage 2 and 24.3% in stage 5	Documentation.		
Lucktar-Flude et al. (2015) (25)	The study aimed to assess how primary care providers apply post-treatment breast cancer survivorship care guidelines. Providers rated the implementation of several guideline recommendations, including fatigue screening and advice.			82 HCPs (59.8% female, mean age 50.1 years, SD 10.8). Professions were family physicians (75.6%) and primary health care nurse practitioners (24.4%).	Guideline usage.
Miller & Kearney (2002) (26)	Nurses' reports were analyzed to evaluate the management of fatigue across various clinical settings. The Institutional Needs Assessment survey, originally developed for pain			204 HCPs. Common age groups were 31-40 years (39%), 20-30 years (27%), and 41-50 years (26%). All were nurses,	Guideline usage, information, and documentation.

	management, was adapted to the context of fatigue.			and the majority were staff nurses (59%) or sisters (30%).	
Milzer et al. (2022) (27)	This longitudinal study assessed patients' perspectives on using a fatigue diary for self-management. The survey evaluated attitudes toward self-management, perceived fatigue management, and the diary's impact. Fatigue was assessed using the EORTC QLQ-FA12.	50 patients (84.0% female, mean age 54.3 years, SD 13.7). Most patients had breast (66.0%), or skin (18.0%) cancer and the majority (60.0%) did not have metastases.	Communication, information, and treatment.		
Nurhesti & Adiputra (2020) (28)	Focusing on HCPs in Indonesia, the study aimed to identify the assessment and treatment practices of CRF through a survey.			112 HCPs (64.3% female). Most common age range 31-40 years (40.2%). Professions were nurses (58.9%) and doctors (27.7%).	Assessment.
Passik et al. (2002) (29)	The study aimed to understand patient barriers to fatigue communication through a survey. The FMBQ assessed	200 patients (57% female, mean age 59.6 years, SD 13.62). Common cancers:	Communication, screening, advice, and treatment.		

	patients' concerns that could hinder fatigue management, while a fatigue intervention checklist explored the recommendations received to alleviate fatigue.	lung (26%), breast (19.9%), and colon (13.8%). Most had stage 3 (28%) or stage 4 (39%).			
Pearson et al. (2015) (30)	Using a survey, HCPs' current practices in assessing and treating CRF were examined. The survey covered practice settings, knowledge of CRF guidelines, screening tools, outcome measures, and obstacles to effective management.			54 HCPs (87% female, mean age 44.3 years, SD 10.27). HCPs were either nurses (81.5%) or doctors (18.5%).	Screening, assessment, and treatment.
Pearson (2017) (31)	This mixed-methods study examined the barriers and facilitators to implementing CRF guidelines. Surveys were distributed to both patients and HCPs. The study was informed by implementation research and the CFIR domains and explored general attitudes toward the CRF guidelines.	68 patients (64% female, mean age 61.1 years, SD 9.0). Common cancers: breast (n=41) and prostate (n=24).	Screening and assessment.		

Pearson et al. (2021) (32)	The feasibility of two ultra-short fatigue screening methods was evaluated, alongside HCPs' fatigue measurement practices and patients' demand for fatigue management. Fatigue documentation and management were assessed through chart reviews. Fatigue levels were measured using the ESAS-r and pictograms in alignment with CAPO CRF guidelines.	430 patients (50.5% female, mean age 62.1 years, SD 15.1). Common cancers: hematology (17.7%), skin (16.7%), head and neck (14.4%), and breast (12.8%).	Documentation.		
Penalba et al. (2019) (33)	Patients' symptom distress, their intent to discuss symptoms, and the actual symptom discussions during oncology visits were investigated through surveys and structured interviews.	94 patients (70.2% female). Most aged 50-69 years (60.2%). Common cancers: gastrointestinal (36.2%), breast (35.1%), and lung (28.7%). Most had stage 2 (23.4%), stage 3 (24.5%), or stage 4 (40.4%).	Communication.		

Rau et al. (2019) (34)	The epidemiology and impact of CRF in Taiwan were explored to enhance physicians' understanding of the symptom. The 'Fatigue Experience Survey', developed based on prior research by Passik et al. (2002) and Shun et al. (2009), assessed the most used CRF interventions. CRF was measured using the ICD-10, BFI-T, and a cancer symptom survey.	1,207 patients (47.8% female, mean age 57.12 years, SD 12.45). Common cancers: breast (16.7%), head and neck (14.5%), and colorectal (11.6%). Most had stage 3 (18.6%) or stage 4 (40%).	Communication and treatment.		
Ruiz et al. (2014) (35)	Unmet needs of HCPs in managing patients receiving targeted therapies for advanced renal cell carcinoma were examined through a survey, which assessed clinical practices and management gaps.			199 HCPs, all physicians.	Treatment.
Schmidt et al. (2016) (36)	This study examined the feasibility of PROMs that are suitable and feasible in German oncology clinics. Patients' PROs	25 patients.	Documentation.		

	were compared with documentation in medical records to assess the possible benefits of PROMs for clinical application.				
Schmidt et al. (2021) (37)	Patient experiences with CRF management in Germany were assessed. Patients were asked, through a survey, whether CRF was assessed, who performed the assessment, which interventions were received, and sources of advice. CRF was measured using a 0-10 NRS.	2,508 patients (48.8% female, mean age 66 years, SD 11.9). Common cancers: breast (9.3%), prostate (8.9%), non-Hodgkin lymphoma (8.4%), kidney (8.3%).	Information, screening, and treatment.		
Schmidt et al. (2022) (38)	A longitudinal study explored cancer patients' knowledge and attitudes toward fatigue, as well as the usefulness of an informational booklet. Fatigue was measured using the EORTC-QLQ-FA12.	50 patients (84% female, mean age 54.3 years, SD 13.7). Common cancers: breast (66%) and skin (18%). 60% had no metastatic disease.	Information.		

Simon et al. (2020) (39)	The study analyzed cancer patients' expectations regarding supportive care. The survey covered different symptoms and the availability of supportive care services.	467 patients (65% female, mean age 61.3 years, SD 11.7). Common cancers: breast (42%) and colorectal (29%). Most had advanced or metastatic disease (65%).	Treatment.	76 HCPs, all oncologists.	Treatment.
Smith et al. (2019) (40)	The prevalence and predictors of cancer-related pain, fatigue, and distress were examined, along with patients' reported experiences with symptom management. The survey, based on the CAHPS Cancer Care Survey, assessed the prevalence of symptoms, related discussions, and the adequacy of clinician-provided support.	2,257 patients (90.8% female, mean age 59.7 years, SD 11.92). Common cancers: breast (82.4%) and colorectal (17.6%). Most had stage 1 (49.1%) or stage 2 (35.8%).	Communication and advice.		
Tarp et al. (2023) (41)	A retrospective study examined the illness trajectory and transitions in metastatic upper gastrointestinal cancer, focusing on palliative care documentation. Nurses' and doctors'	63 patients (46% female). Most aged 71-80 years (49%). Common cancers: pancreatic (n=35) and gastric	Documentation.		

	notes were reviewed to identify symptoms leading to admission and those recorded in specific nursing forms.	(n=12). All had metastatic disease.			
Tolotti et al. (2021) (42)	This mixed-methods study explored patients' and nurses' perceptions of CRF and its management. Patients completed a questionnaire on received fatigue management from nurses. Fatigue management was also evaluated by reviewing clinical records. Semi-structured interviews and focus groups with patients and nurses further examined fatigue experiences. Fatigue levels were assessed using the BFI.	71 patients (45.1% female, mean age 65.87 years, SD 14). Most had hematologic cancer (52.1%).	Communication, screening, assessment, advice, and documentation.		
van Ryn et al. (2014) (43)	Predictors of supportive care quality in colorectal cancer patients were examined, with particular attention to bowel problems, pain, fatigue, depression, and other symptoms. The	1,109 male patients. Most aged under 65 (42.4%) or 65-79 years (42%). All had colorectal cancer, with 35.2%	Communication, screening, advice, and treatment.		

	survey assessed whether HCPs inquired about these symptoms, discussed them, and provided appropriate support.	in stage 1 and 25.1% in stage 2.			
Walling et al (2013) (44)	Non-hospice supportive care was assessed in a national veteran sample through medical record abstraction, evaluating whether appropriate care was provided for each clinical scenario.	719 patients (2.8% female, mean age 66.2 years, SD 10.3). Common cancers: colorectal (37%), lung (33.2%), and pancreatic (29.8%).	Screening.		
Zucca et al. (2016) (45)	A survey explored screening for physical and emotional symptoms across medical oncology centers. Patients reported their perceptions of HCPs' screening behaviors for fatigue, pain, and emotional distress	716 patients (57% female, mean age 61.8 years, SD 11.8). Common cancers: breast (33%) and colorectal (19%).	Screening.		
Zucca et al. (2017) (46)	The proportion of oncology outpatients reporting provider-initiated discussions across multiple domains of patient-centered care was examined through a	244 patients (59% female, mean age 61.6 years, SD 11.9). Common cancers:	Screening.		

	survey, along with factors related to unmet patient preferences	breast (26%), colorectal (20%), and lung (13%).			
Quantitative intervention studies					
Berry et al. (2011) (47)	This RCT evaluated the impact of the ESRA-C on the likelihood of discussing symptoms and QoL issues during consultations, using audio recordings of all sessions.	Control group:<br>333 patients (46.8% female, mean age 54 years, range 18-86). Common cancers: leukemia (17.1%), lymphoma (14.1%), genitourinary (12.3%), and gastrointestinal (12.0%).<br><br>Intervention group:<br>327 patients (45.3% female, mean age 54 years, range 18-89). Common cancers: lymphoma (16.2%), gastrointestinal (12.2%), and genitourinary (11.6%).	Communication.	202 HCPs (56.4% female, 32.6% aged over 55 years). Most were nurses or nurse practitioners (34.2%), attending physicians (34.2%), or residents or fellows (14.4%).	Communication.

Carpenter et al. (2007) (48)	Using a combined longitudinal prospective and retrospective design, this study assessed patient and provider responses to a computerized symptom assessment system. Medical records were reviewed for symptom documentation, and patients were interviewed about symptom discussions HCPs. Surveys were conducted with HCPs to assess the interventions' impact on symptom managements	80 patients (35% female, mean age 57 years, SD 14). Common cancers: sarcoma (35%), gastrointestinal (31%), and prostate (18%).	Communication	8 HCPs, either nurses (n=4) or oncologists (n=4).	Documentation.
Hilarius et al. (2008) (49)	In this sequential cohort design, HRQL assessments were incorporated as a routine part of outpatient chemotherapy. Patients reported whether specific HRQL topics were discussed with nurses, and medical records were reviewed to identify related management activities,	Control group:<br>108 patients (72% female, mean age 55 years). Common cancers: breast (60%) and colorectal (19%).<br><br>Intervention group:	Communication.		

	such as the provision of advice and referrals.	111 patients (68% female, mean age 57 years). Common cancers: breast (46%) and colorectal (26%).			
Marino et al. (2020) (50)	The study introduced PROs in clinical practice, aiming to improve quality of life and symptom reporting. A medical chart review assessed the agreement between patients' and physicians' reports on several symptoms. Fatigue was measured using the EORTC QLQ-C30.	Control group:<br>119 patients (41.2% female, median age 67 years, range 27-84). Common cancers: colorectal (35.3%), lung (21%), pancreatic (11.8%), and genitourinary (10.1%).<br><br>Intervention group:<br>119 patients (41.2% female, median age 67 years, range 27-84). Common cancers: colorectal (35.3%), lung (21%), pancreatic (11.8%), and genitourinary (10.1%).	Documentation.		

Mark (2008) (51)	The aim was to evaluate the PACE-System, an electronic tool for symptom screening and reporting in oncology. Medical charts were reviewed to assess whether the rate of symptom assessments increased after the implementation of PACE.	Control group:<br>100 patients (69% female, mean age 62.25 years, SD 12.5). Common cancers: breast (39.8%) and lung (16.3%).<br><br>Intervention group:<br>100 patients (52.5% female, mean age 60.3 years, SD 11.7). Common cancers: breast (24%) and lung (22%).	Documentation.		
Nicklasson et al. (2013) (52)	The study examined how incorporating individual HRQL data into clinical practice influenced consultations and clinical decision-making. Audio recordings of consultations and medical charts were analyzed to determine whether fatigue was addressed, and	Control group:<br>87 patients (39% female, median age 67 years, range 46-85). All had lung cancer; most had advanced disease.<br><br>Intervention group:	Communication and documentation.		

	which assessment methods and treatments were initiated.	84 patients (37% female, median age 68 years, range 42-89). All had lung cancer; most had advanced disease.			
Tian et al. (2017) (53)	The implementation study, based on the PARiHS framework, assessed outcomes of evidence-based CRF management, including changes in nursing education, procedures, and CRF management practices. Outcomes were evaluated through interviews with HCPs, patient surveys, and medical record reviews			In the first study site: 18 female HCPs (mean age 31.00 years, SD 6.97). All were nurses.<br><br>The second study site: 21 HCPs, all female nurses (mean age 31.33 years, SD 7.80).	Guideline usage, information, screening, assessment, treatment
Wang et al. (2018) (54)	The project aimed to promote evidence-based CRF management using the JBI PACES and GRiP tools. Evaluated outcomes were related to HCP education and the provision of CRF management to patients. These outcomes were examined	30 cancer patients.	Screening, assessment, and advice.		

	through patient interviews and medical record reviews				
Qualitative studies					
Bradford et al. (2023) (55)	The aim of this study was to explore the lived experiences of fatigue in patients with myeloproliferative neoplasms through focus groups and interviews. Patients discussed the impact of fatigue on their lives and shared strategies to alleviate it.	23 patients (78.2% female, median age 58.74 years, range 31.96–76.23). All had myeloproliferative neoplasms.	Communication.		
Browne et al. (2011) (56)	This longitudinal study used in-depth interviews to explore the psychosocial challenges faced by colorectal cancer patients and how they were managed in primary and specialist care. Interviews were conducted at diagnosis and followed up 12 months later. The interview guide included questions about	24 patients (62.5% female). Most common age range: 50-70 years (n=12). All had colorectal cancer, with the majority in Dukes stage B (n=9) or C (n=7).	Communication.		

	the role of various HCPs in managing psychosocial issues				
Charles et al. (2021) (57)	The goal of this study was to explore factors influencing the use of interventions for CRF through semi-structured interviews. Patients discussed the onset of CRF, relief strategies, expectations, and management, with fatigue severity rated using a 10-point NRS.	15 female patients (mean age 60.3 years, range 45–81). All had breast cancer, with 60% in stage 2.	Communication, assessment, advice, and treatment.		
Corbett et al. (2016) (58)	The study used focus groups to assess whether the SRM is a useful framework for conceptualizing CRF in cancer survivors. The interview guide, based on a study by Barsevick et al. (2001), included questions about CRF experiences, its meaning, and strategies for relieving CRF.	18 patients (10 female, mean age 59.83 years, SD 10.34). Common cancers: breast (n=8), prostate (n=3), and stomach (n=2).	Communication, information, and assessment.		

Dolgoy et al. (2020) (59)	This study surveyed head and neck cancer survivors to analyze their descriptions and perceptions of CRF. Questions addressed CRF experiences, personal definitions, and lived experiences.	25 patients (10 female, mean age 61.8 years, SD 6.8). All had head and neck cancer, with the majority in stage 4 (n=18).	Communication and information.		
Erickson et al. (2013) (60)	Semi-structured interviews were conducted to identify fatigue self-care strategies used by patients undergoing radiotherapy. The interviews also assessed the effectiveness of these strategies and explored how patients learned about fatigue management. Fatigue was measured using the BFI.	29 patients (62% female, mean age 58.8 years, SD 10.1). Common cancers: breast (n=9) and prostate (n=4).	Communication.		
Fitch et al. (2008) (61)	In-depth interviews explored how cancer patients cope with CRF, focusing on self-initiated management strategies and their effectiveness. Participants described the	31 patients (16 female, mean age 56.3 years, range 30–78). Common cancers: breast (n=10) and colon (n=9).	Communication, information, and treatment.		

	onset of CRF, its impact on daily life, and strategies for relieving it.				
Glaus et al. (2002) (62)	This study used focus groups to explore cancer patients' perceptions of HCPs' awareness of fatigue and how it is addressed. It also assessed the usability of available fatigue-related information.	13 patients.	Communication, advice, and treatment.		
Horne et al. (2014) (63)	This study used semi-structured interviews to investigate psychosocial supportive care for hematopoietic stem cell transplant patients. Participants discussed and provided interventions, their details, and referral processes.			34 HCPs, of whom 32 nurses and 2 specialty doctors.	Assessment, advice, and information.
Iguchi et al. (2022) (64)	An observational study investigated patient-reported fatigue in Japanese patients receiving androgen receptor-axis-targeted therapies for castration-resistant prostate cancer. Semi-structured interviews explored the burden of	8 male participants (ages 58–76 years). All had prostate cancer.	Communication.		

	fatigue, symptom experience, its impact on daily activities, communication HCPs, and barriers to discussing fatigue. Fatigue was measured using the BFI.				
John (2010) (65)	This study examined self-care strategies employed by lung cancer patients to enhance or maintain their QoL. Semi-structured interviews explored participants' experiences during and after treatment, focusing on QoL perceptions and the self-care practices they used.	10 patients (4 female, mean age 60 years, range 48–78). All had lung cancer, with most in stage 3 (n=6)	Advice.		
Krok-Schoen et al. (2019) (66)	Through focus groups, this study explored symptoms experienced during chemotherapy, patient-clinician communication, and communication barriers among Hispanic breast cancer patients	16 female patients (mean age 57.4 years, SD 10.2). All had breast cancer.	Communication.	5 HCPs (60% female, mean age 48.4 years, SD 11.5). HCPs worked as nurses or nurse practitioners (80%), or physicians (20%).	Communication, information, and advice.

Martin et al. (2021) (67)	This study used semi-structured interviews to explore HCPs' knowledge and use of interventions for CRF. The interview guide, grounded in Horkheimer's critical theory, addressed barriers and needs related to CRF management.			20 HCPs (75% female, median age 36.5 years, range 29-58). Most HCPs were oncologists: 35% medical oncologists, radiation and surgical oncologists (both 15%) or nurses (15%).	Communication, treatment, barriers, and facilitators.
Morgan et al. (2014) (68)	This study explored how African American women manage fatigue during breast cancer treatment through semi-structured interviews. Questions addressed fatigue perception, its impact, and strategies for managing it.	10 female patients (mean age 51.0 years, SD 10.19). All had breast cancer, with half in stage 2.	Information and treatment.		
O'Neill et al. (2023) (69)	Unstructured interviews were conducted to document the experiences of thyroid cancer survivors in their own words.	20 patients (65% female, median age 54 years, range 14–77). All had thyroid cancer, with 80% in stage 1.	Communication and advice.		

Pearson et al. (2017) (31)	This mixed-methods study examined the barriers and facilitators to implementing CRF guidelines. Surveys were distributed to both patients and HCPs. The study was informed by implementation research and the CFIR domains and explored general attitudes toward the CRF guidelines.	68 patients (64% female, mean age 61.1 years, SD 9.0). Common cancers: breast (n=41), prostate (n=24).	Communication, screening, assessment, and advice.	20 HCPs, either working as nurses (n=12) or as doctors (n=8).	Barriers and facilitators
Pearson et al. (2023) (70)	The focus of this study was to assess barriers and strategies for implementing a CRF guideline. Interviews and focus groups explored current practices, as well as HCPs' and patients' preferences for CRF management. The interview guide was based on the CAPO CRF guidelines and the CFIR domains.	5 patients (60% female, median age 59 years, range 43–65). Cancer types: breast (40%), melanoma, bladder, and abdominal cancer (all 20%).	Communication, information, and screening.	21 HCPs, of whom the majority were nurses (n=17). The remaining HCPs (n=4) were specialists, among which were 1 pain specialist, 2 radiation oncologists, and 1 medical oncologist.	Information, assessment, advice, treatment, and barriers.
Pertl et al. (2014) (71)	This study aimed to gain insights into why communication about CRF remains inadequate. It analyzed patients'	73 patients (80% female, mean age 51 years, SD 11.5). Common cancers: breast	Communication, information, and assessment.		

	additional comments from a quantitative questionnaire that explored their beliefs and coping strategies regarding CRF.	(28.8%), ovarian (21.9%), and lymphoma (19.1%).			
Piredda et al. (2007) (72)	By replicating and expanding on the study by Glaus et al. (2002), this study aimed to explore Italian patients' and nurses' views on educational resources for CRF. Through focus groups, participants shared their educational needs and preferences and barriers to discussing CRF.	7 patients (5 female, mean age 64.3 years, SD 5.4). Female patients had breast cancer, male patients had melanoma and prostate cancer.	Information and advice.	6 HCPs (age range 24-34 years). All HCPs were nurses, working as staff nurses (n=4) or nurse managers (n=2).	Information, advice, treatment, and barriers.
Porock & Juenger (2004) (73)	This study aimed to describe and explore the experience of fatigue in patients undergoing biotherapy and to identify their informational needs through interviews.	10 patients (3 female, age range 30–62 years).	Information.		
Robertsen & Skär (2021) (74)	The purpose of this study was to explore oncology nurses' experiences in addressing CRF with male cancer			9 HCPs, all female nurses with a median age of 47 years, range 36-56.	Information, treatment, and facilitators.

	patients. Semi-structured interviews examined strategies for focusing on the emotional aspects of CRF and helping patients find meaning in their condition.				
Siegel et al. (2012) (75)	The goal of this study was to understand the fatigue attributions of advanced cancer patients aged 55 years or older. In-depth interviews explored the attributions of fatigue, how they were formed, and their consequences.	35 patients (54% female, mean age 67 years, range 56-88). Common cancers: lymphoma (17%) and multiple myeloma (11%). All had stage 3 or stage 4 disease.	Communication and information.		
Snyder et al. (2010) (76)	This study aimed to identify the topics that patients and clinicians consider relevant for use in PROs in clinical practice. Semi-structured interviews provided insights into which topics were discussed during patient visits and which patients would like to discuss and track using a questionnaire	41 patients (51.2% female, mean age 63.8 years, SD 12.56). Cancer types: breast (51.2%) and prostate (48.8%).	Communication.	15 HCPs (33.4% female, mean age 48.85 years, SD 7.14). All HCPs were physicians, specializing in medical oncology (46.7%), radiation oncology (33.3%), or in	Communication.

				urology or surgery (combined 20.0%).	
Spichiger et al. (2022) (77)	The study aimed to explore how cancer chemotherapy patients perceive fatigue. Interviews with participants focused on fatigue communication with HCPs, self-care strategies, and the perceived effectiveness of these strategies.	19 patients (11 female, mean age 45 years, range 23–77). Common cancers: breast (n=7), colorectal (n=6), lymphoma (n=5), and lung (n=1). 8 patients had palliative treatment goals.	Communication, information, screening, and advice.		
Tang et al. (2018) (78)	This study focused on analyzing recorded healthcare encounters to better understand the symptom experience of advanced pancreatic cancer patients.	37 patients (56.76% female, mean age 66 years), all with advanced pancreatic cancer.	Communication and screening.		
Tolotti et al. (2021) (42)	This mixed-methods study explored patients' and nurses' perceptions of CRF and its management. Patients completed a questionnaire on received fatigue management from nurses. Fatigue management was also evaluated by	71 patients (45.1% female, mean age 65.87 years, SD 14). Most had hematological cancer (52.1%).	Information and advice.	15 HCPs, all nurses. Most were female (n=12) and were within the age range of 25-45 years.	Screening, assessment, advice, treatment, documentation, and facilitators.

	reviewing clinical records. Semi-structured interviews and focus groups with patients and nurses further examined fatigue experiences. Fatigue levels were assessed using the BFI.				

Note: Only the data pertinent to the scope of this review are reported. Some studies may include additional methods or participant groups that are not presented in the table.

BFI, Brief Fatigue Inventory; BFI-T, Brief Fatigue Inventory-Taiwan; CAHPS, Consumer Assessment of Healthcare Providers and Systems; CAPO, Canadian Association of Psychosocial Oncology; CFIR, Consolidated Framework for Implementation Research; CRF, cancer-related fatigue; EORTC QLQ-C30, European Organization for Research and Treatment of Cancer Core Quality of Life; EORTC QLQ-FA13, European Organization for Research and Treatment of Cancer Quality of Life Fatigue module; ESAS-r, Edmonton Symptom Assessment System Revised; ESRA-C, Electronic Self-Report Assessment–Cancer; FACT-F, Functional Assessment of Cancer Therapy-Fatigue subscale; FIS, Fatigue Impact Scale; FMBQ, Fatigue Management Barriers Questionnaire; GP, general practitioner; GRiP; Getting Research into Practice; HCP, healthcare professional; HRQL, health-related quality of life; ICD-10, International Classification of Diseases-Tenth Revision; IQR, interquartile range; JBI PACES, Joanna Briggs Institute Practical Application of Clinical Evidence System; MFSI-SF, Multidimensional Fatigue Symptom Inventory-Short Form; NCCN, National Comprehensive Cancer Network; NRS, numeric rating scale; PACE, Patient Assessment, Care and Education; PARiHS, Promoting Action on Research Implementation in Health Services; PFS, Piper Fatigue Scale; PRO, patient-reported outcome; PROM, patient-reported outcome measure; QLACS, Quality of Life in Adult Cancer Survivors; QoL, quality of life; RCT, randomized controlled trial; SD, standard deviation; SRM, self-regulation model; SRQ, Symptom Representation Questionnaire; VAS, visual analogue scale.
