# Supplementary material for: The gap between guidelines and clinical practice in cancer-related fatigue management: a mixed-methods systematic review
Source: J Cancer Surviv. 2025 Jul 18;20(Suppl 1):1220–39. doi: 10.1007/s11764-025-01866-6 (PMC13676525; doi:10.1007/s11764-025-01866-6)
Supplement: Supplementary file 4 [file 11764_2025_1866_MOESM4_ESM.pdf]

Supplementary Materials: File 4

The Gap Between Guidelines and Clinical Practice in Cancer-Related Fatigue Management: A Mixed-Methods Systematic Review

Journal of Cancer Survivorship

Marijn Witziers[a], Anne Looijmans[a], Ning Jia[a], & Mariët Hagedoorn[a]

[a], Department of Health Psychology, University Medical Centre Groningen, University of Groningen, the Netherlands

Correspondence: Marijn Witziers

Email: m.witziers@umcg.nl

Reference list of all included studies

1. Al Maqbali M, Gracey J, Dunwoody L, Rankin J, Hacker E, Hughes C. Healthcare professionals knowledge on cancer-related fatigue: A cross-sectional survey in Oman. Nurs Health Sci. 2020;22(3):732-40. https://doi.org/10.1111/nhs.12720

2. Alcalde-Castro MJ, Soto-Perez-de-Celis E, Covarrubias-Gomez A, Sanchez-Roman S, Quiroz-Friedman P, Navarro-Lara A, et al. Symptom assessment and early access to supportive and palliative care for patients with advanced solid tumors in Mexico. J Palliat Care. 2020;35(1):40-5. https://doi.org/10.1177/0825859719834920

3. Berry DL, Blumenstein BA, Halpenny B, Wolpin S, Fann JR, Austin-Seymour M, et al. Enhancing patient-provider communication with the electronic self-report assessment for cancer: a randomized trial. J Clin Oncol. 2011;29(8):1029-35. https://doi.org/10.1200/jco.2010.30.3909

4. Blaney JM, Lowe-Strong A, Rankin-Watt J, Campbell A, Gracey JH. Cancer survivors' exercise barriers, facilitators and preferences in the context of fatigue, quality of life and physical activity participation: a questionnaire-survey. Psychooncology. 2013;22(1):186-94. https://doi.org/10.1002/pon.2072

5. Bock M, Moore D, Hwang J, Shumay D, Lawson L, Hamolsky D, et al. The impact of an electronic health questionnaire on symptom management and behavior reporting for breast cancer survivors. Breast Cancer Res Treat. 2012;134(3):1327-35. https://doi.org/10.1007/s10549-012-2150-1

6. Borneman T, Piper BF, Sun VC, Koczywas M, Uman G, Ferrell B. Implementing the Fatigue Guidelines at one NCCN member institution: process and outcomes. J Natl Compr Canc Netw. 2007;5(10):1092-101. https://doi.org/10.6004/jnccn.2007.0090

7. Bradford A, Young K, Whitechurch A, Burbury K, Pearson EJM. Disabled, invisible and dismissed—The lived experience of fatigue in people with myeloproliferative neoplasms. Cancer Reports. 2023;6(1). https://doi.org/doi:10.1002/cnr2.1655

8. Browne S, Dowie A, Mitchell E, Wyke S, Ziebland S, Campbell N, Macleod U. Patients' needs following colorectal cancer diagnosis: where does primary care fit in? Br J Gen Pract. 2011;61(592):e692-9. https://doi.org/10.3399/bjgp11X606582

9. Butt Z, Wagner LI, Beaumont JL, Paice JA, Straus JL, Peterman AH, et al. Longitudinal screening and management of fatigue, pain, and emotional distress associated with cancer therapy. Support Care Cancer. 2008;16(2):151-9. https://doi.org/10.1007/s00520-007-0291-2

10. Cardoso F, Rihani J, Harmer V, Harbeck N, Casas A, Rugo HS, et al. Quality of life and treatment-related side effects in patients with HR+/HER2− advanced breast cancer: Findings from a multicountry survey. The Oncologist. 2023;28(10):856-65. https://doi.org/10.1093/oncolo/oyad207

11. Carpenter JS, Rawl S, Porter J, Schmidt K, Tornatta J, Ojewole F, et al. Oncology outpatient and provider responses to a computerized symptom assessment system. Oncol Nurs Forum. 2008;35(4):661-9. https://doi.org/10.1188/08/onf.661-669

12. Carreira H, Williams R, Dempsey H, Bhaskaran K. Recording of patients' mental health and quality of life-related outcomes in primary care: a cross-sectional study in the UK. BMJ Open. 2022;12(12):e066949. https://doi.org/doi:10.1136/bmjopen-2022-066949

13. Charles C, Di Meglio A, Arnedos M, Arvis J, Baciarello G, Blanchard P, et al. QualFatigue study: which factors influence the use of specific interventions for breast cancer survivors with fatigue? A cross-sectional exploratory study. Support Care Cancer. 2021;29(8):4827-34. https://doi.org/10.1007/s00520-021-06040-z

14. Cheville AL, Shen T, Chang M, Basford JR. Appropriateness of the treatment of fatigued patients with stage IV cancer. Support Care Cancer. 2013;21(1):229-33. https://doi.org/10.1007/s00520-012-1515-7

15. Chouaïd C, Colin P, Maloisel F, Mitry E, Zelek L, Le Calvé P. Regards croisés 2009: enquête menée auprès de patients atteints de cancer et de professionnels de santé sur la perception de la fatigue et de l’anémie chimio-induite. Oncologie. 2010;12(3):239-44. https://doi.org/10.1007/s10269-010-1869-8

16. Collins S, de Vogel-Voogt E, Visser A, van der Heide A. Presence, communication and treatment of fatigue and pain complaints in incurable cancer patients. Patient Educ Couns. 2008;72(1):102-8. https://doi.org/10.1016/j.pec.2008.02.011

17. Corbett T, Groarke A, Walsh JC, McGuire BE. Cancer-related fatigue in post-treatment cancer survivors: application of the common sense model of illness representations. BMC Cancer. 2016;16(1):919. https://doi.org/10.1186/s12885-016-2907-8

18. de Ligt KM, Heins M, Verloop J, Smorenburg CH, Korevaar JC, Siesling S. Patient-reported health problems and healthcare use after treatment for early-stage breast cancer. Breast. 2019;46:4-11. https://doi.org/10.1016/j.breast.2019.03.010

19. Diaz N, Menjon S, Rolfo C, Garcia-Alonso P, Carulla J, Magro A, et al. Patients' perception of cancer-related fatigue: results of a survey to assess the impact on their everyday life. Clin Transl Oncol. 2008;10(11):753-7. https://doi.org/10.1007/s12094-008-0282-x

20. Dillon E, Kelly J, Aifc. The status of cancer fatigue on the Island of Ireland: AIFC professional and interim patient surveys. Oncologist. 2003;8 Suppl 1:22-6. https://doi.org/10.1634/theoncologist.8-suppl_1-22

21. Dolgoy ND, O'Krafka P, McNeely ML. Cancer-related fatigue in head and neck cancer survivors: Energy and functional impacts. Cancer Treat Res Commun. 2020;25:100244. https://doi.org/10.1016/j.ctarc.2020.100244

22. Donovan HS, Ward S. Representations of fatigue in women receiving chemotherapy for gynecologic cancers. Oncol Nurs Forum. 2005;32(1):113-6. https://doi.org/10.1188/05.Onf.113-116

23. Erickson JM, Spurlock LK, Kramer JC, Davis MA. Self-care strategies to relieve fatigue in patients receiving radiation therapy. Clin J Oncol Nurs. 2013;17(3):319-24. https://doi.org/10.1188/13.Cjon.319-324

24. Fitch MI, Mings D, Lee A. Exploring patient experiences and self-initiated strategies for living with cancer-related fatigue. Can Oncol Nurs J. 2008;18(3):124-40. https://doi.org/10.5737/1181912x184124131

25. Glaus A, Frei IA, Knipping C, Ream E, Browne N. [Attitude of cancer patients to fatigue: patient attitude in Switzerland and England]. Pflege. 2002;15(5):187-94. https://doi.org/10.1024/1012-5302.15.5.187

26. Harrington SE, Fisher MI, Lee JQ, Cohn J, Malone D. Knowledge regarding cancer-related fatigue: a survey of physical therapists and individuals diagnosed with cancer. Physiother Theory Pract. 2022:1-10. https://doi.org/10.1080/09593985.2022.2056554

27. Heins MJ, Korevaar JC, Rijken PM, Schellevis FG. For which health problems do cancer survivors visit their General Practitioner? Eur J Cancer. 2013;49(1):211-8. https://doi.org/10.1016/j.ejca.2012.07.011

28. Hershman D, Calhoun E, Zapert K, Wade S, Malin J, Barron R. Patients' perceptions of physician-patient discussions and adverse rvents with cancer therapy. Arch Drug Inf. 2008;1(2):70-8. https://doi.org/10.1111/j.1753-5174.2008.00011.x

29. Hilarius DL, Kloeg PH, Gundy CM, Aaronson NK. Use of health-related quality-of-life assessments in daily clinical oncology nursing practice: a community hospital-based intervention study. Cancer. 2008;113(3):628-37. https://doi.org/10.1002/cncr.23623

30. Hingst H, Stutz U, Buchholz I. Tumorbedingte fatigue - Prozessevaluation eines Seminars der Universitatsmedizin Greifswald. Pflege. 2021;34(1):41-9. https://doi.org/10.1024/1012-5302/a000778

31. Horne B, Gilleece M, Jackson G, Snowden JA, Liebersbach S, Velikova G, Wright P. Psychosocial supportive care services for haematopoietic stem cell transplant patients; a service evaluation of three UK transplant centres. European Journal of Cancer Care. 2014;23(3):349-62. https://doi.org/doi:10.1111/ecc.12160

32. Iguchi T, Nakamura Y, Akiyama T, Chand K, Yu E. Descriptive study on burden and communication of fatigue among castration-resistant prostate cancer patients in Japan. Curr Med Res Opin. 2022;38(3):417-26. https://doi.org/10.1080/03007995.2021.2006534

33. James S, Wright P, Scarlett C, Young T, Jamal H, Verma R. Cancer-related fatigue: results from patient experience surveys undertaken in a UK regional cancer centre. Support Care Cancer. 2015;23(7):2089-95. https://doi.org/10.1007/s00520-014-2565-9

34. Jenkins V, Catt S, Banerjee S, Gourley C, Montes A, Solis-Trapala I, et al. Patients' and oncologists' views on the treatment and care of advanced ovarian cancer in the U.K.: results from the ADVOCATE study. Br J Cancer. 2013;108(11):2264-71. https://doi.org/10.1038/bjc.2013.223

35. John LD. Self-care strategies used by patients with lung cancer to promote quality of life. Oncol Nurs Forum. 2010;37(3):339-47. https://doi.org/10.1188/10.Onf.339-347

36. Krok-Schoen JL, Fern, ez K, Unzeitig GW, Rubio G, Paskett ED, Post DM. Hispanic breast cancer patients' symptom experience and patient-physician communication during chemotherapy. Support Care Cancer. 2019;27(2):697-704. https://doi.org/doi:10.1007/s00520-018-4375-y

37. La Verde G, Arienti D. [Cancer-related fatigue in hematological cancer: hematologist's and patient's view]. Recenti Prog Med. 2002;93(9):463-9.

38. Leung E, Fineberg H, Larsen T, Yaver M, Foo A, Ma J, et al. An observational study evaluating the impact on prostate patient outcomes and experiences when radiation therapists use a standard grading system tool to assess and document treatment-related toxicities and interventions. Journal of Medical Imaging and Radiation Sciences. 2022;53(3):444-52. https://doi.org/doi:10.1016/j.jmir.2022.06.004

39. Luctkar-Flude M, Aiken A, McColl MA, Tranmer J, Langley H. Are primary care providers implementing evidence-based care for breast cancer survivors? Can Fam Physician. 2015;61(11):978-84.

40. Maloisel F, Mitry E, Zelek L, Chouaid C, Colin P, Le Calve P. Regards croisés 2009: perception of cancer patients and health professionals about their relations in the management of cancer and fatigue. Oncologie. 2010;12(8):495-501. https://doi.org/10.1007/s10269-010-1914-7

41. Marino D, Baratelli C, Guida G, Cleopatra Turco CG, Lacidogna G, Sperti E, et al. Impact of adoption of patient-reported outcomes in clinical practice on the accuracy of symptom reporting in medical records of cancer patients. Recenti Progressi in Medicina. 2020;111(12):740-8. https://doi.org/doi:10.1701/3509.34965

42. Mark TL, Fortner B, Johnson G. Evaluation of a tablet PC technology to screen and educate oncology patients. Support Care Cancer. 2008;16(4):371-8. https://doi.org/10.1007/s00520-007-0312-1

43. Martin E, Zingarello A, Di Meglio A, Baciarello G, Matias M, Charles C, et al. A qualitative evaluation of the use of interventions to treat fatigue among cancer survivors: A healthcare provider's view. Eur J Cancer Care (Engl). 2021;30(2):e13370. https://doi.org/10.1111/ecc.13370

44. Miller M, Kearney N. Institutional management of cancer-related fatigue: a comparison of clinical specialties. Eur J Oncol Nurs. 2002;6(1):45-53. https://doi.org/10.1054/ejon.2001.0138

45. Milzer M, Steindorf K, Reinke P, Schmidt ME. The cancer patients' perspective on feasibility of using a fatigue diary and the benefits on self-management: results from a longitudinal study. Support Care Cancer. 2022;30(12):10213-21. https://doi.org/10.1007/s00520-022-07397-5

46. Morgan PD, Tyler I, Fogel J, Barnett K. African American women share 'real talk' stories about fatigue related to breast cancer treatment. Abnf j. 2014;25(4):116-22.

47. Nicklasson M, Elfström ML, Olofson J, Bergman B. The impact of individual quality of life assessment on psychosocial attention in patients with chest malignancies: a randomized study. Support Care Cancer. 2013;21(1):87-95. https://doi.org/s10.1007/s00520-012-1496-6

48. Nurhesti POY, Adiputra IN. Assessment and management of cancer-related fatigue: Health care providers' perceptions. Enferm Clin. 2020;30:86-9. https://doi.org/10.1016/j.enfcli.2020.07.018

49. O'Neill CJ, Carlson MA, Rowe CW, Fradgley EA, Paul C. Hearing the voices of Australian thyroid cancer survivors: Qualitative thematic analysis of semistructured interviews identifies unmet support needs. Thyroid. 2023. https://doi.org/10.1089/thy.2023.0080

50. Passik SD, Kirsh KL, Donaghy K, Holtsclaw E, Theobald D, Cella D, et al. Patient-related barriers to fatigue communication: initial validation of the fatigue management barriers questionnaire. J Pain Symptom Manage. 2002;24(5):481-93. https://doi.org/10.1016/s0885-3924(02)00518-3

51. Pearson EJ, Denehy L, Edbrooke L. Identifying strategies for implementing a clinical guideline for cancer-related fatigue: a qualitative study. BMC Health Services Research. 2023;23(1):395. https://doi.org/10.1186/s12913-023-09377-9

52. Pearson EJ, Drosdowsky A, Edbrooke L, Denehy L. Exploring the use of two brief datigue screening tools in cancer outpatient clinics. Integr Cancer Ther. 2021;20:1534735420983443. https://doi.org/10.1177/1534735420983443

53. Pearson EJ, Morris ME, McKinstry CE. Cancer-related fatigue: a survey of health practitioner knowledge and practice. Support Care Cancer. 2015;23(12):3521-9. https://doi.org/10.1007/s00520-015-2723-8

54. Pearson EJ, Morris ME, McKinstry CE. Cancer related fatigue: implementing guidelines for optimal management. BMC health services research. 2017;17. https://doi.org/10.1186/s12913-017-2415-9

55. Penalba V, Deshields TL, Klinkenberg D. Gaps in communication between cancer patients and healthcare providers: symptom distress and patients' intentions to disclose. Support Care Cancer. 2019;27(6):2039-47. https://doi.org/10.1007/s00520-018-4442-4

56. Pertl MM, Quigley J, Hevey D. 'I'm not complaining because I'm alive': barriers to the emergence of a discourse of cancer-related fatigue. Psychol Health. 2014;29(2):141-61. https://doi.org/10.1080/08870446.2013.839792

57. Piredda M, De Marinis MG, Rocci L, Gualandi R, Tartaglini D, Ream E. Meeting information needs on cancer-related fatigue: an exploration of views held by Italian patients and nurses. Support Care Cancer. 2007;15(11):1231-41. https://doi.org/10.1007/s00520-007-0240-0

58. Porock D, Juenger JA. Just go with the flow: a qualitative study of fatigue in biotherapy. Eur J Cancer Care (Engl). 2004;13(4):356-61. https://doi.org/10.1111/j.1365-2354.2004.00497.x

59. Rau KM, Shun SC, Chiou TJ, Lu CH, Ko WH, Lee MY, et al. A nationwide survey of fatigue in cancer patients in Taiwan: An unmet need. Jpn J Clin Oncol. 2020;50(6):693-700. https://doi.org/10.1093/jjco/hyaa038

60. Robertsen IL, Skär L. Oncology nurses' experiences of meeting with men with cancer-related fatigue: a qualitative study. Scand J Caring Sci. 2021;35(1):252-9. https://doi.org/10.1111/scs.12841

61. Ruiz JN, Belum VR, Creel P, Cohn A, Ewer M, Lacouture ME. Current practices in the management of adverse events associated with targeted therapies for advanced renal cell carcinoma: a national survey of oncologists. Clin Genitourin Cancer. 2014;12(5):341-7. https://doi.org/10.1016/j.clgc.2014.04.001

62. Schmidt H, Merkel D, Koehler M, Flechtner HH, Sigle J, Klinge B, et al. PRO-ONKO-selection of patient-reported outcome assessments for the clinical use in cancer patients-a mixed-method multicenter cross-sectional exploratory study. Support Care Cancer. 2016;24(6):2503-12. https://doi.org/10.1007/s00520-015-3055-4

63. Schmidt ME, Bergbold S, Hermann S, Steindorf K. Knowledge, perceptions, and management of cancer-related fatigue: The patients' perspective. Support Care Cancer. 2021;29(4):2063-71. https://doi.org/10.1007/s00520-020-05686-5

64. Schmidt ME, Milzer M, Weiss C, Reinke P, Grapp M, Steindorf K. Cancer-related fatigue: benefits of information booklets to improve patients' knowledge and empowerment. Support Care Cancer. 2022;30(6):4813-21. https://doi.org/10.1007/s00520-022-06833-w

65. Siegel K, Lekas HM, Maheshwari D. Causal attributions for fatigue by older adults with advanced cancer. J Pain Symptom Manage. 2012;44(1):52-63. https://doi.org/10.1016/j.jpainsymman.2011.07.013

66. Simon H, Ganem G, Touboul C, Lhomel C, Morere JF. Patient perspectives on supportive care in cancer: Results of the Calista 2 study. Eur J Cancer Care (Engl). 2020;29(6):e13299. https://doi.org/10.1111/ecc.13299

67. Smith TG, Troeschel AN, Castro KM, Arora NK, Stein K, Lipscomb J, et al. Perceptions of patients with breast and colon cancer of the management of cancer-related pain, fatigue, and emotional distress in community oncology. J Clin Oncol. 2019;37(19):1666-76. https://doi.org/10.1200/jco.18.01579

68. Snyder CF, Jensen RE, Geller G, Carducci MA, Wu AW. Relevant content for a patient-reported outcomes questionnaire for use in oncology clinical practice: Putting doctors and patients on the same page. Qual Life Res. 2010;19(7):1045-55. https://doi.org/10.1007/s11136-010-9655-z

69. Spichiger E, Rieder E, Muller-Frohlich C, Kesselring A. Fatigue in patients undergoing chemotherapy, their self-care and the role of health professionals: a qualitative study. Eur J Oncol Nurs. 2012;16(2):165-71. https://doi.org/10.1016/j.ejon.2011.05.002

70. Tang CC, Draucker C, Tejani M, Von Ah D. Symptom experiences in patients with advanced pancreatic cancer as reported during healthcare encounters. Eur J Cancer Care (Engl). 2018;27(3):e12838. https://doi.org/10.1111/ecc.12838

71. Tarp MS, Danielsen AK, Rosenberg J. Palliative needs and illness trajectory associated with metastatic upper gastrointestinal cancer. Danish Medical Journal. 2023;70(2).

72. Tian L, Yang Y, Sui W, Hu Y, Li H, Wang F, et al. Implementation of evidence into practice for cancer-related fatigue management of hospitalized adult patients using the PARIHS framework. PLoS One. 2017;12(10):e0187257. https://doi.org/10.1371/journal.pone.0187257

73. Tolotti A, Bonetti L, Pedrazzani C, Bianchi M, Moser L, Pagnucci N, et al. Nursing management of fatigue in cancer patients and suggestions for clinical practice: a mixed methods study. BMC Nurs. 2021;20(1):182. https://doi.org/10.1186/s12912-021-00699-9

74. van Ryn M, Phelan SM, Arora NK, Haggstrom DA, Jackson GL, Zafar SY, et al. Patient-reported quality of supportive care among patients with colorectal cancer in the Veterans Affairs Health Care System. J Clin Oncol. 2014;32(8):809-15. https://doi.org/10.1200/jco.2013.49.4302

75. Walling AM, Tisnado D, Asch SM, Malin JM, Pantoja P, Dy SM, et al. The quality of supportive cancer care in the veterans affairs health system and targets for improvement. JAMA Intern Med. 2013;173(22):2071-9. https://doi.org/10.1001/jamainternmed.2013.10797

76. Wang N, Yang Z, Miao J, Mi X, Liu S, Stern C, et al. Clinical management of cancer-related fatigue in hospitalized adult patients: A best practice implementation project. JBI Database System Rev Implement Rep. 2018;16(10):2038-49. https://doi.org/10.11124/jbisrir-2017-003769

77. Zucca A, Sanson-Fisher R, Waller A, Carey M, Boadle D. The first step in ensuring patient-centred quality of care: ask the patient. Eur J Cancer Care (Engl). 2017;26(1). https://doi.org/10.1111/ecc.12435

78. Zucca A, Sanson-Fisher R, Waller A, Carey M, Boyes AW, Proietto A. Does screening for physical and psychosocial symptoms vary between medical oncology treatment centres? Psychooncology. 2016;25(5):521-8. https://doi.org/10.1002/pon.3948
